# Supplementary material for: A Quantitative Chemotherapy Genetic Interaction Map Reveals Factors Associated with PARP Inhibitor Resistance
Source: Cell Rep. Author manuscript; Available in PMC 2018 May 4. (PMC5935461; doi:10.1016/j.celrep.2018.03.093)
Supplement: 1 [file NIHMS962087-supplement-1.pdf]

Cell Reports, Volume *23*

Supplemental Information

A Quantitative Chemotherapy Genetic Interaction Map Reveals Factors Associated with PARP Inhibitor Resistance

Hsien-Ming Hu, Xin Zhao, Swati Kaushik, Lilliane Robillard, Antoine Barthelet, Kevin K. Lin, Khyati N. Shah, Andy D. Simmons, Mitch Raponi, Thomas C. Harding, and Sourav Bandyopadhyay

SUPPLEMENTAL FIGURES

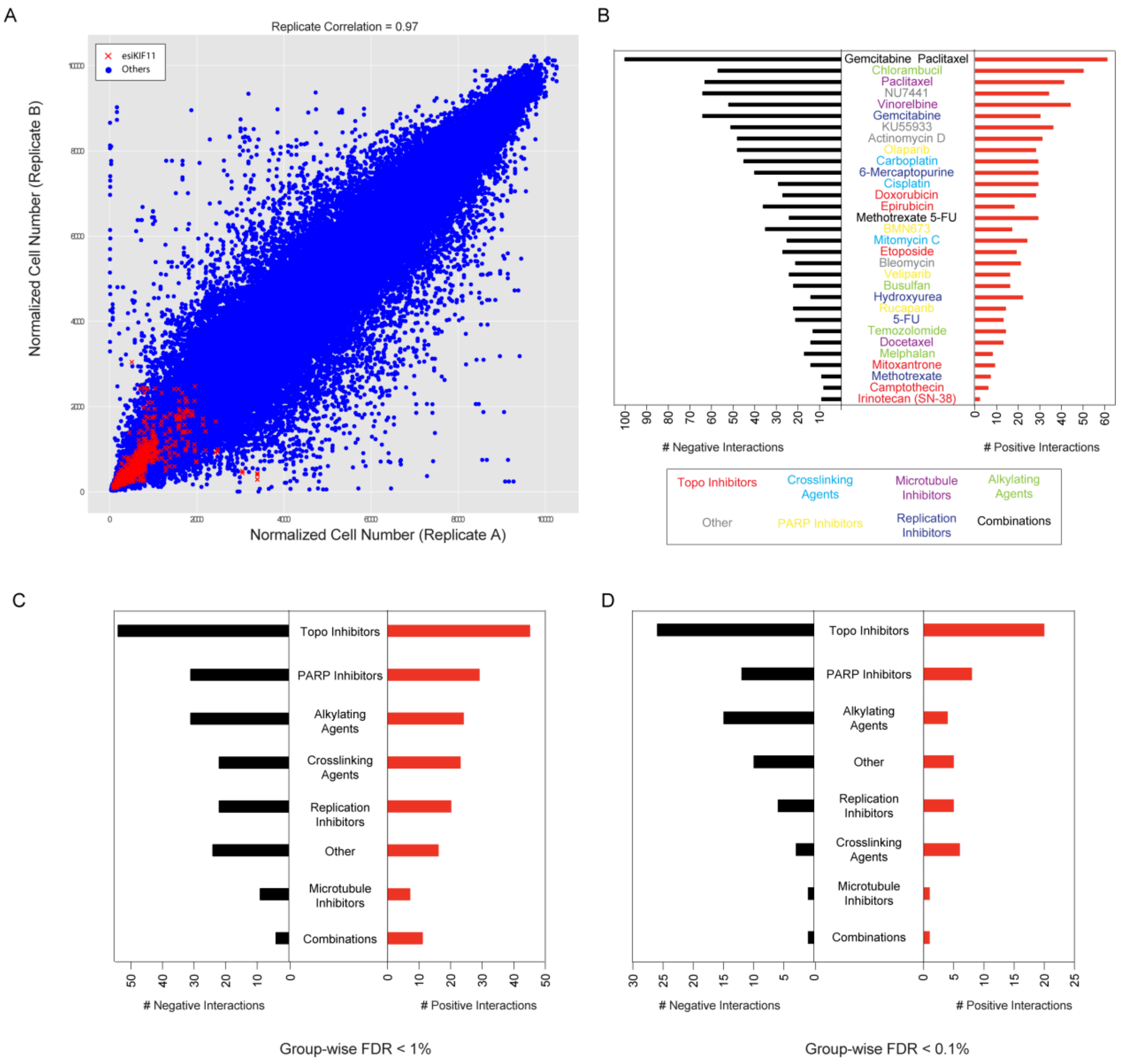

Figure S1. Properties of the chemical-genetic interaction map, Related to Figure 1 and 2. (A) Comparison of cell number between technical replicates shown. Red crosses indicate cell numbers in the context of knockdown of the essential gene KIF11. Pearson correlation among replicates shown. **(B)** Number of high confidence interaction per drug. Interactions are considered if they have a score greater than 3 (positive) or less than -3 (negative) corresponding to a 1% FDR. **(C-D)** Number of interactions based on common interactions with drugs in the same class. The median of interactions of a given gene with all drugs in a class was compared to a permuted background to derive a False Discovery Rate (FDR). Number of interactions at an FDR (C) less than 1% or (D) less than 0.1%

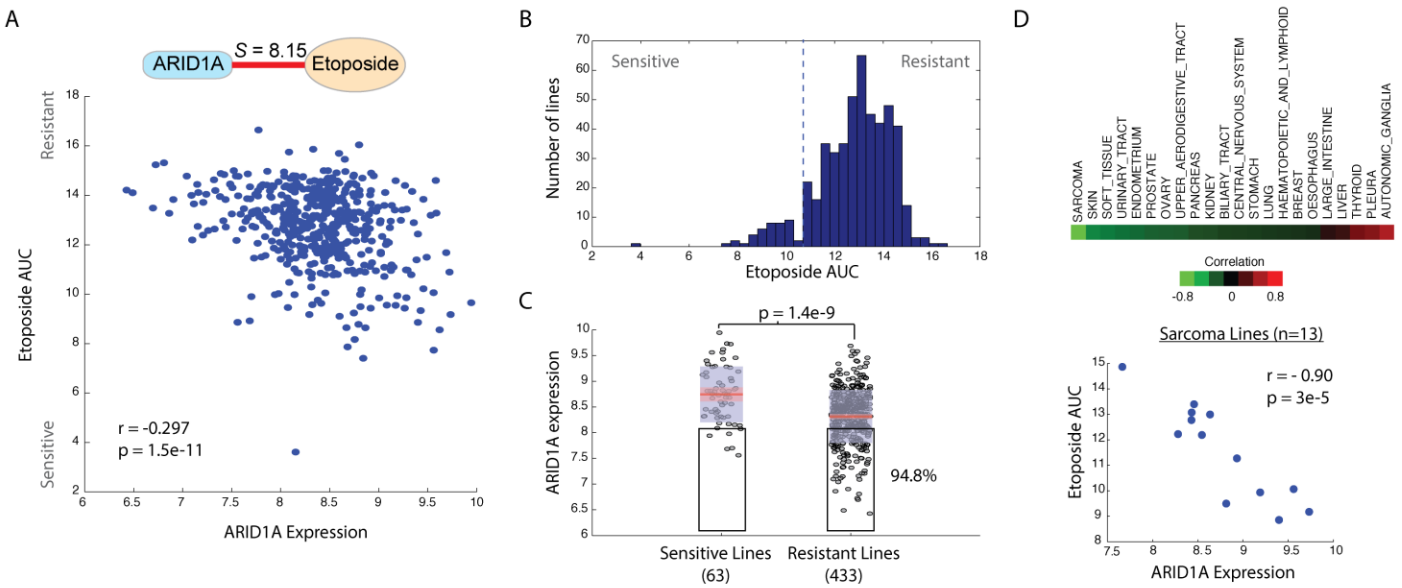

Figure S2. ARID1A loss is associated with etoposide resistance as predicted by the map, Related to Figure 2. (A) Correlation of ARID1A expression and etoposide sensitivity across 496 cancer cell lines based on area under the curve (AUC) analyis of drug sensitivity in the CTRP dataset. **(B)** Histogram of sensitivity to Etoposide across 496 lines. Dotted line represents an AUC of 11 which is used to separate 63 sensitive versus 433 resistant lines based on an approximately bimodal distribution. **(C)** ARID1A expression in sensitive versus resistant lines. Lines with expression < 8 are outlined and 94.8% of the cell lines that have this level of expression or lower are considered drug resistant. **(D)** Correlation analysis of ARID1A expression with etoposide AUC across subsets of cell lines with the indicated lineages. Scatter plot of expression and AUC for sarcoma lines shown. P-values based on pearson correlation except for (C) where it is based on a two-tailed t-test. Box plots are medians +/- s.d.

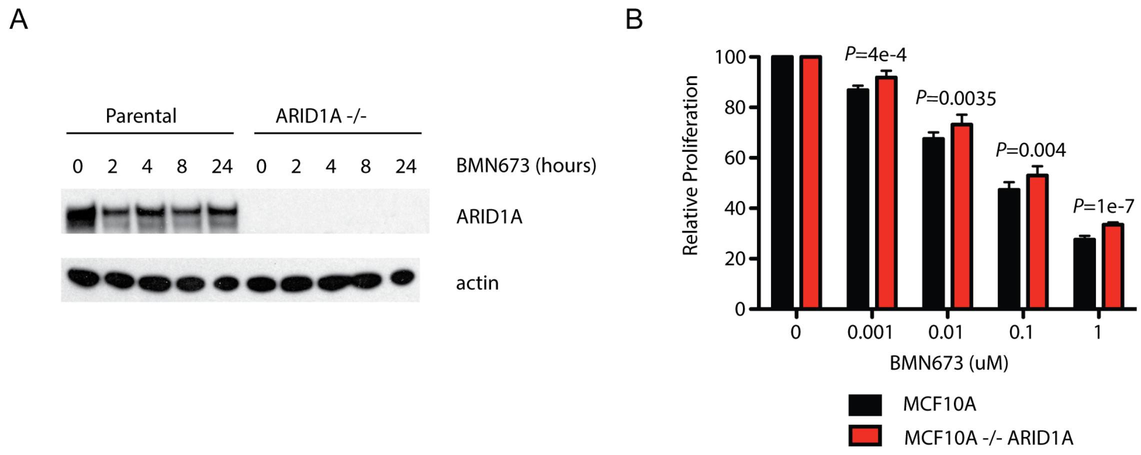

Figure S3. ARID1A deletion leads to PARP inhibitor resistance, Related to Figure 5. (A) Verification of ARID1A loss in isogenic MCF10A parental and ARID1A -/- cells by immunoblot. **(B)** Sensitivity to BMN673 of ARID1A null cells compared to parental MCF10A.

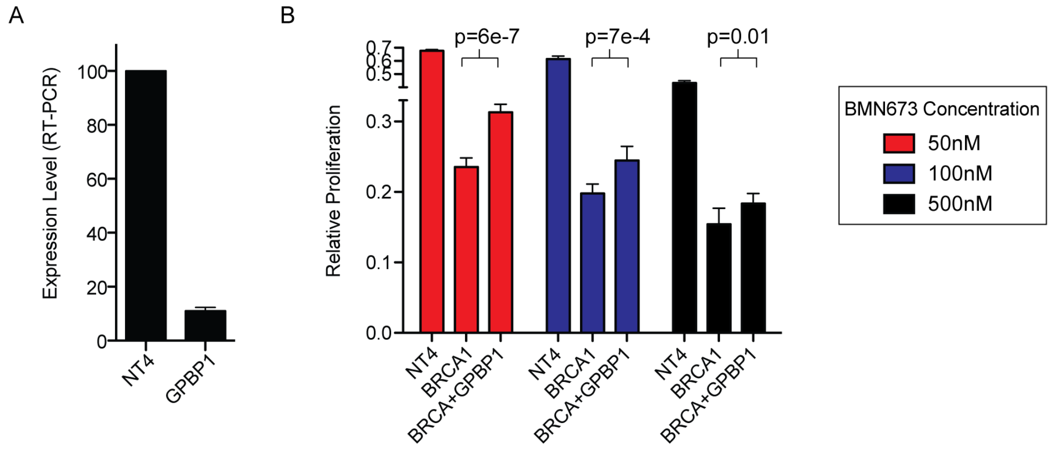

Figure S4. GPBP1 knockdown and effects on BMN673 sensitivity, Related to Figure 6. (A) RNA from MCF10A cells transfected with either siNT4 or siGPBP1 was subjected to RT-PCR analysis for GPBP1 expression. **(B)** Proliferation of MCF10A cells trasnfected with siRNAs targeting the indicated gene or non-targeting (NT4) and then treated with the indicated dose of BMN673 for 3 days.

SUPPLEMENTAL EXPERIMENTAL PROCEDURES

Immunofluorescence microscopy

MCF10A cells were grown on glass coverslip pre-coated with polylysine. To induce DNA damage repair foci the cells were treated with 0.5mM BMN673 for 4 or 8 hours. Cells were fixed with 4% paraformaldehyde in PBS for 10 min, permeablized with 0.3% Triton for 10 min, and blocked with 3% BSA in PBS for 40 min at room temperature. Cells were then incubated with antibodies against gH2AX (JBW301, Upstate) or RAD51 (SC-8349, Santa Cruz) at 4°C overnight, followed by the incubation with species-specific Alexa488 or Alexa647-conjugated secondary antibodies (Thermofisher) for 1hr at room temperature. Coverslips were mounted on the slides in Vectashield containing DAPI for nuclear counterstain. Images were acquired with a confocal microscope (Zeiss LSM 780) equipped with a 63x water immersion objective and a CCD camera, using the ZEN software. Automated foci counting was done using the Foci Counter (The Bioimaging Center, University of Konstanz) plugin in ImageJ. At least 150 cells were scored for each experiment, and each experiment was repeated three times.

siRNA transfection

SMARTpool ON-TARGETplus siRNAs targeting BRCA1(L-003461-00-0005), ARID1A (L-017263-00-0005), GPBP1 (L-014236-02-0005) and non-targeting control siNT4 were purchased from GE Dharmacon. siRNAs were transfected into cells using Lipofectamine RNAiMax transfection reagent (ThermoFisher) according to the manufacturer's instruction. The cells were transfected in 384-well plates at a final siRNA concentration of 20nM. After an initial 24h period of gene knockdown, drug sensitivity was determined by adding the indicated drugs for 72h.

Drug combination screening

Cells were seeded onto 384-well plates (1,000 cells/well) 18 hours prior to treatment with drug either singly or in combination with another drug. A pair of drugs is combined in a series of one DMSO and five

or seven concentrations centered on the IC_{50} dose, resulting in a 6 × 8 escalating combination dose matrix using a robotic liquid handler. After a 72 hour drug treatment, cell proliferation was determined by staining with Hoescht 33342 (5ug/ml) and cell number counted using a Thermo CellInsight High Content microscope. Synergistic effects between different drug combos was measured by determination of synergy score using comparison to Loewe additive model with Chalice Analyzer (Zalicus Inc. Cambridge, MA). Combination Index values were calculated using CompuSyn (www.compusyn.com).

Cell line drug sensitivity prediction

A total of 568 genes were mapped between the chemical-genetic interaction map and gene expression data obtained from (https://portals.broadinstitute.org/ccle/home). Expression data was normalized to a median of 0 across cell lines. Sensitivity for each drug was defined by its area under the curve, or AUC (Basu et al., 2013). To predict the sensitivity of a given cell line for a particular drug in an unsupervised fashion, the sign of the chemical-genetic interaction score for each gene in the map was multiplied by the normalized expression of that gene and this product summed over all genes in the map. Hence, for each cell line the predicted AUC for drug *d* ($pAUC_d$) is:

$$pAUC_d = -\sum_{g \in G} e_g * sign(s_{gd})$$

Where G is the set of all genes in the map for drug d, s_{gd} is the score of drug d with gene g and e_g is the normalized expression of g in this cell line. This procedure rewards for cases where the expression and interaction scores are consistent, i.e. when both expression and score is negative or both expression and score are positive, and in both these cases will predict drug sensitivity. This procedure was performed at differing cutoffs of the interaction map based on the absolute value of the score. For each drug a *pAUC* was computed for each cell line and this value was then correlated with the published AUC.

Reverse transcription PCR (RT-qPCR)

2.5 mg of total RNA was reverse transcribed using murine Moloney leukemia virus (MMLV) reverse transcriptase, with oligo dT and template switching oligos as primers for cDNA synthesis. Real-time PCR with SYBR green detection was performed using an ABI Prism 7700 thermocycler (Applied Biosystem). The house keeping genes HPRT-1 and b2M was used as control for normalization. A ΔΔCq method was used to process the data for the target gene (GPBP1) to calculate relative gene expression normalized to house keeping genes.

RNAseq analysis

RNA extraction from MCF10A cells was performed with RNeasy mini kit (Qiagen). The samples were treated sequentially with Baseline-ZERO DNase and Ribo-ZERO RNA removal kit (Epicentre) to deplete DNA and rRNA. 35ng of rRNA-depleted sample was used as input for library preparation using ScriptSeq v2 library preparation kit (Epicentre) according to the manufacturer's instructions. All libraries were indexed with Illumina barcodes for multiplexing. The quality of the library preparations was assessed on the 2100 Bioanalyzer (Agilent) using a High Sensitivity DNA Chip. The quantitation of the libraries was performed using the Qubit dsDNA HS Assay kit (Life technology). Sequencing was performed on an Illumina HiSeq 4000 for single-ended 50 base paired reads in duplicate on Illumina Hi-Seq 2000 at the Center for Advanced Technology at the University of California, San Francisco. Raw sequencing reads were analyzed using FASTQC for quality control and adapters were filtered using cutadapt (Martin, 2017). The filtered raw reads were pseudoaligned and quantification of the transcripts was performed with Kallisto (Bray et al., 2016) using GRC38 reference human transcriptome. Transcript abundance estimates were converted to gene level estimates using an R package Tximport (Soneson et al., 2015). Differential expression analysis between sets of conditions was performed using DESeq2 and averaged over both replicates (Anders and Huber, 2010). Analysis of the enrichment of functional gene groups and pathways among differentially expressed genes was performed using gene-set enrichment analysis (GSEA) (Subramanian et al., 2005). Enrichment plots were calculated based on ranking of differentially

expressed genes between different conditions. RNA-seq data are available under GEO:GSE101904 (https://www.ncbi.nlm.nih.gov/geo/).

Clinical trial data analysis

Tumor samples were tested for mutations in a panel of 322 genes using FoundationOne test from Foundation Medicine (Waltham, MA). In the ARIEL-2 trial (NCT01891344) 154 patients were selected that were wild type in HR pathways genes including *BRCA1*, *BRCA2* and *CDK12*. Clinical characteristics were taken from Swisher et al (Swisher et al., 2017). ARIEL-2 was approved by the institutional review board at each study site and was done in accordance with the Declaration of Helsinki and Good Clinical Practice Guidelines of the International Conference on Harmonisation. Patients provided written informed consent before participation.

Statistical analysis

All error bars are s.d. unless otherwise noted. All P-values based on two-tailed t-test.

Supplemental References

Anders, S., and Huber, W. (2010). Differential expression analysis for sequence count data. Genome biology *11*, R106.

Basu, A., Bodycombe, N.E., Cheah, J.H., Price, E.V., Liu, K., Schaefer, G.I., Ebright, R.Y., Stewart, M.L., Ito, D., Wang, S., *et al.* (2013). An interactive resource to identify cancer genetic and lineage dependencies targeted by small molecules. Cell *154*, 1151-1161.

Bray, N.L., Pimentel, H., Melsted, P., and Pachter, L. (2016). Near-optimal probabilistic RNA-seq quantification. Nature biotechnology *34*, 525-527.

Martin, M. (2017). Cutadapt removes adapter sequences from high-throughput sequencing reads. EMBnet.

Soneson, C., Love, M.I., and Robinson, M.D. (2015). Differential analyses for RNA-seq: transcript-level estimates improve gene-level inferences. F1000Research *4*, 1521.

Subramanian, A., Tamayo, P., Mootha, V.K., Mukherjee, S., Ebert, B.L., Gillette, M.A., Paulovich, A., Pomeroy, S.L., Golub, T.R., Lander, E.S., *et al.* (2005). Gene set enrichment analysis: a knowledge-based approach for interpreting genome-wide expression profiles. Proceedings of the National Academy of Sciences of the United States of America *102*, 15545-15550.

Swisher, E.M., Lin, K.K., Oza, A.M., Scott, C.L., Giordano, H., Sun, J., Konecny, G.E., Coleman, R.L., Tinker, A.V., O'Malley, D.M., *et al.* (2017). Rucaparib in relapsed, platinum-sensitive high-grade ovarian carcinoma (ARIEL2 Part 1): an international, multicentre, open-label, phase 2 trial. The Lancet Oncology *18*, 75-87.
