# Supplementary material for: A Quantitative Chemotherapy Genetic Interaction Map Reveals Factors Associated with PARP Inhibitor Resistance
Source: Cell Rep. Author manuscript; Available in PMC 2018 May 4. (PMC5935461; doi:10.1016/j.celrep.2018.03.093)
Supplement: 7 [file NIHMS962087-supplement-7.pdf]

Resource

Cell Reports

A Quantitative Chemotherapy Genetic Interaction Map Reveals Factors Associated with PARP Inhibitor Resistance

Graphical Abstract

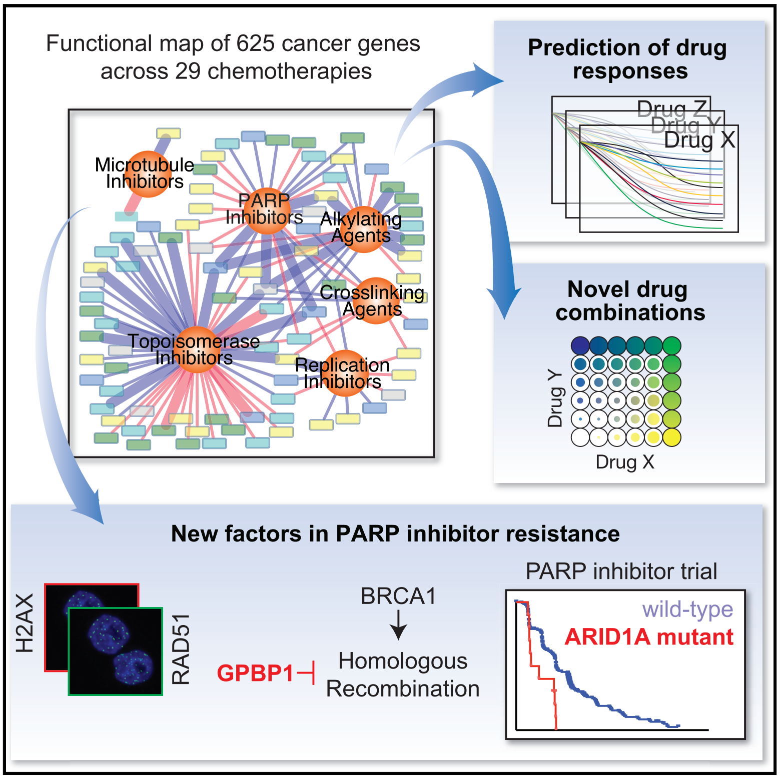

Authors

Hsien-Ming Hu, Xin Zhao, Swati Kaushik, ..., Mitch Raponi, Thomas C. Harding, Sourav Bandyopadhyay

Correspondence

sourav.bandyopadhyay@ucsf.edu

In Brief

Hu et al. map the impact of knockdown of 625 cancer and DNA repair genes on the cellular response to every class of chemotherapy. This map can be used to predict drug responses and identify synergistic drug combinations, and it reveals two factors, ARID1A and GPBP1, whose loss contributes to PARP inhibitor resistance.

Highlights

- Chemotherapy genetic interaction map covering 625 genes and 29 DNA-damaging agents
- Map predicts cancer cell line drug responses and synergistic drug combinations
- ARID1A loss confers PARP inhibitor resistance in cells and ovarian cancer patients
- GPBP1 loss confers PARP inhibitor resistance via regulation of HR gene expression

Data and Software Availability

GSE101904

Hu et al., 2018, Cell Reports *23*, 918–929
April 17, 2018 © 2018 The Authors.
https://doi.org/10.1016/j.celrep.2018.03.093

CellPress

OPEN ACCESS
CellPress

Cell Reports
Resource

A Quantitative Chemotherapy Genetic Interaction Map Reveals Factors Associated with PARP Inhibitor Resistance

Hsien-Ming Hu,[1] Xin Zhao,[1] Swati Kaushik,[1] Lilliane Robillard,[2] Antoine Barthelet,[1] Kevin K. Lin,[2] Khyati N. Shah,[1] Andy D. Simmons,[2] Mitch Raponi,[2] Thomas C. Harding,[2] and Sourav Bandyopadhyay[1,3,*]
[1]Bioengineering and Therapeutic Sciences, Helen Diller Family Comprehensive Cancer Center and Institute for Computational Health Sciences. University of California, San Francisco, San Francisco, CA 94158, USA
[2]Clovis Oncology, Inc., Boulder, CO 80301, USA
[3]Lead Contact
*Correspondence: sourav.bandyopadhyay@ucsf.edu
https://doi.org/10.1016/j.celrep.2018.03.093

SUMMARY

Chemotherapy is used to treat most cancer patients, yet our understanding of factors that dictate response and resistance to such drugs remains limited. We report the generation of a quantitative chemical-genetic interaction map in human mammary epithelial cells charting the impact of the knockdown of 625 genes related to cancer and DNA repair on sensitivity to 29 drugs, covering all classes of chemotherapy. This quantitative map is predictive of interactions maintained in other cell lines, identifies DNA-repair factors, predicts cancer cell line responses to therapy, and prioritizes synergistic drug combinations. We identify that ARID1A loss confers resistance to PARP inhibitors in cells and ovarian cancer patients and that loss of GPBP1 causes resistance to cisplatin and PARP inhibitors through the regulation of genes involved in homologous recombination. This map helps navigate patient genomic data and optimize chemotherapeutic regimens by delineating factors involved in the response to specific types of DNA damage.

INTRODUCTION

Chemotherapy is given to the vast majority of cancer patients and used based on average responses rather than personalized decisions (Barcenas et al., 2014). Limited improvements in survival by the use of chemotherapy also highlight the need to develop drugs and make better use of existing drugs (Early Breast Cancer Trialists' Collaborative Group, 2005). Furthermore, choosing from multiple possible chemotherapy options can complicate clinical decision making. Therefore, optimizing the use of chemotherapies is a significant and pressing challenge in precision oncology. Chemotherapies commonly target the heightened proliferation resulting from unrestrained cell-cycle and DNA-damage checkpoints present in cancer cells, but their narrow therapeutic window results in the dose-limiting toxicities common with these agents. While tumors that harbor specific alterations in DNA-repair genes such as *BRCA1*, *BRCA2*, and *ERCC1* are more responsive to certain chemotherapies (Byrski et al., 2012; Olaussen et al., 2006), our knowledge of relevant biomarkers for chemotherapy remains limited. Therefore, understanding the impact that tumor mutations have on modifying drug responses can lead to more efficient use of chemotherapy.

Recent advances in genomics have led to a dramatic increase in the rate of discovery of altered genes in patient tumors. This explosion in knowledge has led to bottlenecks at the level of a functional understanding of tumor genomes, a key step in therapeutic development. Chemical-genetic interaction maps can aid in elucidating roles for genetic events in cancers by causally linking them to drug sensitivity (Martins et al., 2015; Muellner et al., 2011). Furthermore, effectively connecting gene alterations with therapeutics will also require clarity regarding the exact mechanism of drug actions, which are often lacking for classical chemotherapeutic agents as well as newly developed drugs targeting DNA repair and processing (Cheung-Ong et al., 2013; Helleday, 2011; Liu et al., 2012; Mitchison, 2012). In the case of PARP inhibitors, their efficacy may be dependent on their ability to trap PARP onto DNA, leading to DNA double-strand breaks during replication rather than blocking the repair of single-strand breaks through enzymatic inhibition of PARP, as initially hypothesized (Helleday, 2011; Murai et al., 2012). It is likely that insights into the mechanisms of action of chemotherapies will need to be combined with an understanding of gene function in order to create predictive models of drug responses in patients.

A key milestone in the field was the discovery that tumor cells that are deficient in *BRCA1* or *BRCA2* are sensitive to PARP inhibitors in a synthetic lethal manner, ultimately leading to approval of these agents for the treatment of ovarian cancer. Mechanistically, this synthetic lethal interaction takes advantage of a deficiency in homologous recombination (HR) caused by *BRCA1/2* mutation that is necessary to repair DNA lesions incurred by PARP inhibition (Bryant et al., 2005; Farmer et al., 2005). With the approval of several PARP inhibitors, both *de novo* and acquired resistance to PARP inhibitors have become an important clinical problem. What appears to be critical for resistance is the restoration of HR that, in some cases, can be attributed to secondary intragenic mutations, which restores BRCA1 or BRCA2 functionality (Norquist et al., 2011). Although additional factors have been reported, little is known

This is an open access article under the CC BY license (http://creativecommons.org/licenses/by/4.0/).

about their relevance to resistance in the clinic (Lord and Ashworth, 2013). Central to emerging mechanisms of resistance is the interplay between two major repair pathways, non-homologous end joining (NHEJ) and HR. In a competitive model between these two pathways, the NHEJ factor TP53BP1 suppresses HR, and TP53BP1 loss restores HR, facilitating PARP inhibitor resistance (Bouwman et al., 2010; Bunting et al., 2010; Chapman et al., 2012). However, *TP53BP1* loss has not been observed in patients, suggesting that additional factors may contribute to the resistant phenotype.

Here, we generate a systematic resource that quantitatively maps the influence of the knockdown of 612 DNA-repair and cancer-relevant genes on the responses to 31 chemotherapeutic agents in breast cancer, covering nearly all major Food and Drug Administration (FDA)-approved chemotherapies. We demonstrate that the map recovers many known modulators of chemosensitivity and is able to link therapies with common mechanisms of action. We show that the map is a predictive tool to computationally infer cancer cell line drug sensitivity and design drug combinations with targeted inhibitors of ATR and APEX1. We also identify ARID1A and GPBP1 as factors whose loss contributes to PARP inhibitor and platinum resistance, a finding that is supported by data from HGSOC patients. This chemical-genetic interaction map can be used to identify factors that dictate responses to chemotherapy and aid in the translation from tumor genomics to therapeutics.

RESULTS

Generation of a Chemotherapy-Based Genetic Interaction Map in Breast Epithelial Cells

We developed a quantitative chemical-genetic interaction mapping strategy to uncover the impact of gene loss on proliferative responses to a panel of approved chemotherapies as well as emerging inhibitors of DNA repair. Beyond common tumor suppressor genes, we focused on genes recurrently deleted in breast and ovarian cancer. We mined The Cancer Genome Atlas (TCGA) studies as well as the METABRIC breast cancer cohort, covering over 3,000 samples to identify a set of over 200 breast and 170 ovarian cancer genes whose deletion occurred with high frequency in these studies (Figure 1A; Table S1) (Cancer Genome Atlas Network, 2012; Cancer Genome Atlas Research Network, 2011; Curtis et al., 2012). We also included nearly all genes known to be involved in DNA repair (n = 134). As a complement, we assembled a collection of 29 distinct compounds encompassing nearly all FDA-approved chemotherapies for breast and ovarian cancer, four PARP inhibitors, and two other targeted therapies (Figure 1B). In addition, we profiled two common drug combinations, for a total of 31 distinct treatments. The map was generated in MCF10A cells, which are immortal, epithelial, diploid, HR competent, and devoid of mutations in known oncogenes (Debnath et al., 2002). By molecular profiling, these cells are receptor-negative or basal-like, a subtype that has been shown to be similar in biology and etiology to high-grade serous ovarian cancer (Cancer Genome Atlas Network, 2012). Knockdowns were performed using endonuclease-prepared siRNAs (esiRNAs), which are enzymatically cleaved long double-stranded RNAs that exist in a pool with high sequence complexity and exhibit fewer off-target effects than synthetic siRNA (small interfering RNA)-based approaches (Kittler et al., 2007). To generate the chemical-genetic interaction map, MCF10A cells were transfected with individual esiRNAs, exposed to either DMSO or drug, and allowed to proliferate for 72 hr before counting. Knockdown of an essential gene, KIF11, was used as positive control in the screen (Figure S1A). Normalized cell numbers from each knockdown in the presence of drug or DMSO were compared to identify differences in proliferation over 8 replicates (4 in each condition), and the significance of this difference was converted into a signed chemical-genetic interaction score (*S*) (Experimental Procedures; Martins et al., 2015). Positive *S* scores indicate that the gene loss caused drug resistance, and negative *S* scores indicate that gene loss induced drug sensitivity that could constitute a synthetic lethal interaction. Analysis of the distribution of scores based on knockdown of GFP as negative control allowed the assignment of false discovery rates (FDRs) of 10%, 5%, and <1% to cutoffs of $S = \pm3$, ±4, and ±5, respectively (Figure 1C). Altogether, we determined quantitative scores for 19,406 gene-drug interactions and identified 1,042 positive and 740 negative interactions at $S = \pm3$, corresponding to a 10% FDR (Table S2). These interactions mapped to a median of 27 positive and 22 negative interactions per drug (Figure S1B).

As a control, we examined the impact of loss of BRCA proteins on sensitivity to PARP inhibitors, a known synthetic lethal interaction (Bryant et al., 2005; Farmer et al., 2005). Loss of BRCA1 or BRCA2 was among the most synthetic lethal with PARP inhibitors in our dataset, including strong interactions with the PARP inhibitor BMN673 (BRCA1 $S = -4.4$; BRCA2 $S = -5.6$). This finding also extended to members of the BRCA pathway, SHFM1 ($S = -2.9$) and PALB2 ($S = -4.9$), which mediate HR as previously reported (Figure 1D) (Buisson et al., 2010; McCabe et al., 2006). We also observed strong synthetic lethal interactions between BRCA1/2- and BRCA-pathway genes and DNA cross-linking agents cisplatin and mitomycin C (BRCA1 with cisplatin, $S = -5.8$; and with mitomycin C, $S = -5.1$) (Figure 1D). Synthetic lethality of BRCA1 with PARP inhibitors is related to the ability of the drug to trap PARP onto chromatin (Murai et al., 2014; Shen et al., 2013). Using the map, we asked whether this trend extends beyond BRCA1 to the entire HR pathway. We examined known genes involved in HR and found that they were also often synthetic lethal with PARP inhibitors in a manner that was related to the degree of PARP trapping onto DNA (Figure 1E). Illustrating this point, the strongest trapper, BMN673, had an average score of -2.4 with 19 known components of HR ($p = 3.1e{-4}$), which was lower than with any other PARP inhibitor. Since these drugs are comparable inhibitors of PARP enzymatic activity, our results indicate that synthetic lethality with loss of components of HR machinery is more dependent on PARP trapping than enzymatic inhibition. Loss of the NHEJ factor TP53BP1 has been shown to cause resistance to PARP inhibitors in several models (Bouwman et al., 2010; Bunting et al., 2010; Chapman et al., 2012). This was also reflected in the chemical-genetic map, with TP53BP1 knockdown conferring resistance to PARP inhibitors (BMN673 $S = 3.3$) and DNA cross-linkers (cisplatin $S = 4.3$) (Figures 1D and 1E). We conclude that the chemical-genetic interaction

OPEN
ACCESS
CellPress

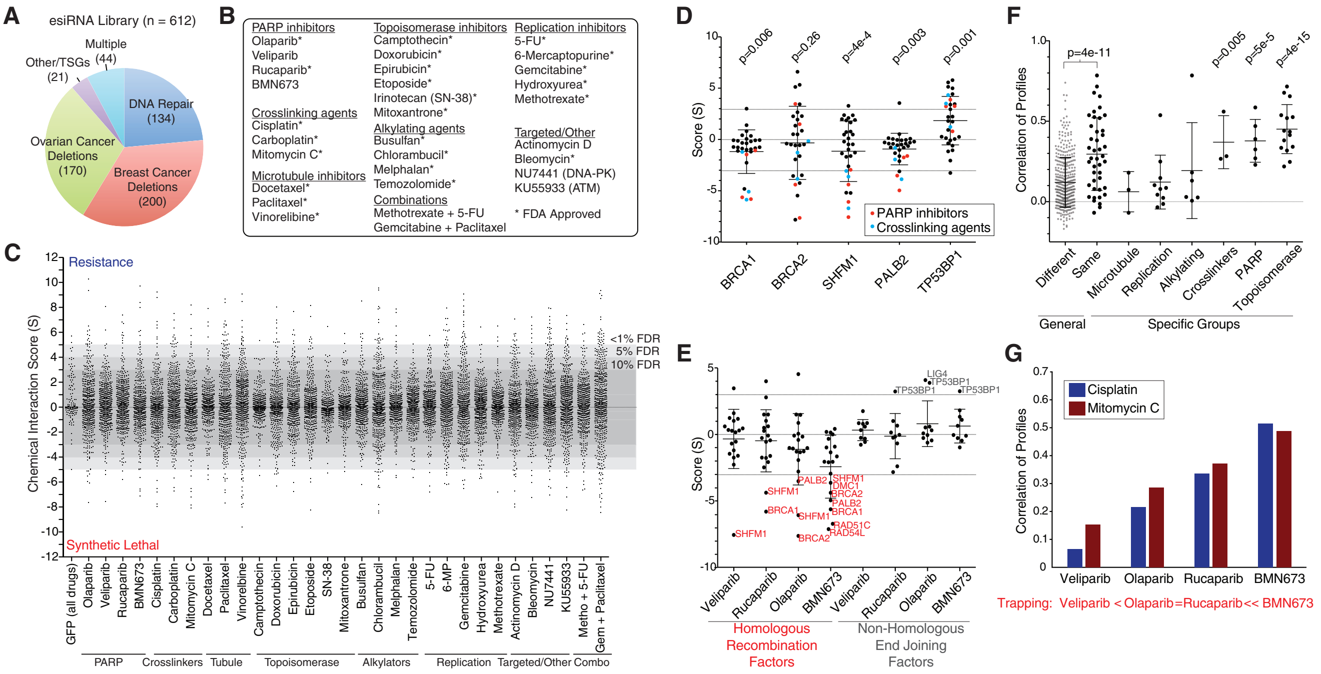

Figure 1. Design of a Chemical-Genetic Interaction Map and Recapitulation of Known Gene and Drug Relationships

(A) Composition of genes selected for the map. TSGs, tumor suppressor genes.

(B) Selection of 31 drugs profiled in this study.

(C) Distribution of chemical genetic interaction scores (*S*) for drugs profiled. Scores of 899 GFP knockdowns across all tested drugs are indicated. FDR cutoffs are based on the percentage of GFP knockdowns falling outside of a given score threshold. Metho, methotrexate; Gem, gemcitabine.

(D) Genetic interactions with BRCA-pathway members BRCA1, BRCA2, SHFM1, and PALB2, as well as the NHEJ factor TP53BP1. Interactions with PARP inhibitors and crosslinking agents are highlighted, and p values represent the significance of differences between these scores and zero, using a t test. Dotted lines represent 10% FDR cutoff.

(E) PARP inhibitor scores with annotated HR and NHEJ factors.

(F) Correlation of interaction profiles among drugs that belong to the same or a different class. For each drug class, pairwise correlations were compared against a background of correlations between drugs from different classes to determine a p value.

(G) Correlation of profiles for PARP inhibitors with two cross-linking agents, cisplatin and mitomycin C. Trapping potency published in Murai et al. (2014). Data indicate mean ± SD.

See also Figure S1 and Tables S1 and S2.

map recapitulates known drivers of chemosensitivity and resistance in a quantitative fashion and is a resource for the identification of potential drivers of the drug response.

Chemical-Genetic Profiles Link Drugs with Similar Mechanisms of Action

While broad classes of chemotherapeutics target various aspects of DNA processing and repair, their exact mechanisms of action are often unclear (Cheung-Ong et al., 2013). Therefore, we asked whether the map could be used to link together drugs based on common mechanisms of action. For a given drug, its profile of chemical interaction scores represents a high-resolution phenotype that can be compared to other drugs. Calculating all-pairwise correlations between drugs revealed that drugs known to operate in the same general class had a higher average correlation of profiles as compared to drugs that were unrelated (Figure 1F, p = 4e−11). Overall, this trend was highest for topoisomerase and PARP inhibitors, as well as DNA cross-linkers, which were all significantly more inter-related compared to background (Figure 1F). For topoisomerase inhibitors, their profiles were highly correlated (mean r = 0.45, p = 4e−15) and exemplified by the shared profiles of topoisomerase II inhibitors, etoposide and doxorubicin (r = 0.65, p = 5e−79). The ability to link drugs with similar mechanisms of action led us to further investigate the mechanism of action of PARP inhibitors. We found a strong correlation of profiles comparing PARP inhibitors with cisplatin and mitomycin C that both work by causing intra-strand crosslinks that block replication (mean r = 0.35, p = 6.9e−7). However, this correlation was highly related to PARP-trapping ability, with the most potent trapper, BMN673, having the highest correlation with cisplatin (r = 0.51) and mitomycin C (r = 0.49) (Figure 1G). Taken together, our results further support the model whereby PARP trapping creates double-strand breaks during replication in a manner similar to that of cisplatin and mitomycin C and that HR is necessary to repair these lesions. Therefore, the genetic interaction map provides a high-resolution means to understand similarities and differences between the mechanisms of action of drugs.

OPEN ACCESS
CellPress

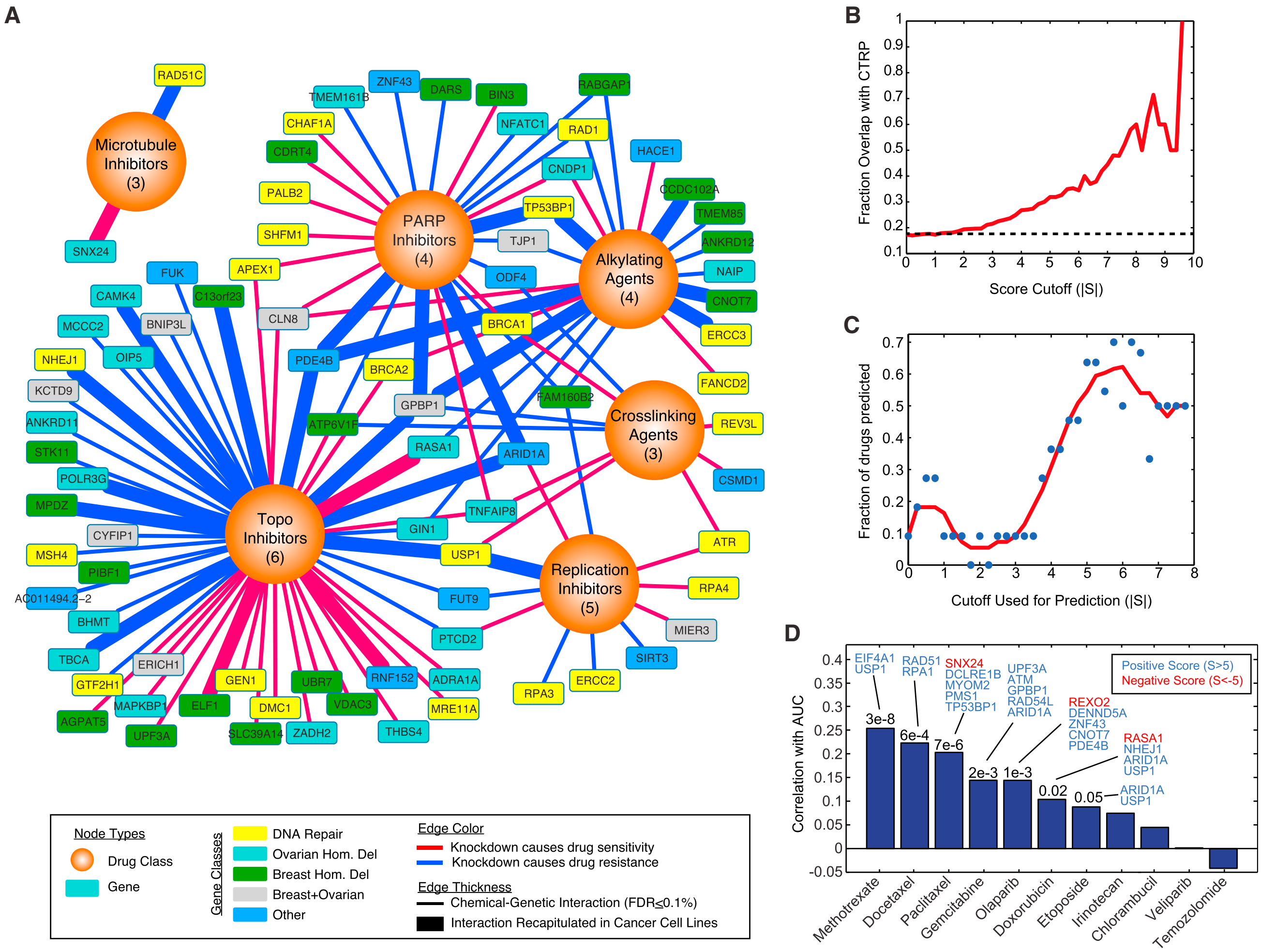

Figure 2. Prediction of Cell Line Responses from the Chemical-Interaction Map

(A) Consensus interaction map based on coordinate responses with drug classes. All interactions shown have an FDR of category association <0.1%. The number of drugs in each category is indicated. Thicker edges represent interactions that are also found across cancer cell line collections ($p < 0.01$).

(B) Overlap with correlation-based chemical-genetic interactions from cancer cell lines. Indicated is the fraction of chemical-genetic interactions at a given score cutoff ($|S|$), where the expression of the gene is also significantly associated with resistance or sensitivity to the same drug across cell lines in the CTRP dataset ($p < 0.01$). Dotted line represents baseline overlap at random (17.3%).

(C) Prediction of cell line responses to 11 drugs overlapping with the CTRP dataset. Cell lines were scored based on the sum of normalized gene expression for all genes in the network at a given cutoff (Experimental Procedures). These drug- and cell-line-specific scores are then correlated with the area under curve (AUC) values reported in the CTRP, and significant predictors are counted ($p \leqslant 0.05$). Red line indicates a sliding average.

(D) Analysis of cell line response predictions based on a score cutoff of 5. For each model, the correlation of predicted AUC versus real AUC is indicated, with accompanying p values when significant. Genes whose expression contributed the most to the prediction accuracy are indicated (Experimental Procedures).

See also Figures S1 and S2 and Table S3.

Prediction of Cancer Cell Line Responses Using the Chemical-Genetic Interaction Map

Based on the similarity of profiles between related drugs, we next sought to combine genetic interactions based on drug class to identify a consensus chemical-genetic interaction map. In this consensus map, a connection between a gene and a compound category reflects a concordance of response across multiple related drugs and compared against a randomly permuted background. At an FDR of 0.1% we identified 125 connections between genes and different drug classes (Figure 2A; Table S3). While connections spanned all major drug classes, topoisomerase inhibitors, PARP inhibitors, and alkylating agents made up the majority of this network, while microtubule inhibitors were under-represented due to the lack of genetic interactions in common across this class of agents (Figures S1C and S1D). Through the analysis of independent chemical entities sharing a common mechanism, this map highlights many potential modifiers of drug responses that are altered in breast and ovarian cancers that may participate in the DNA-damage response.

The ability of the chemical-genetic interaction map to identify causal genetic relationships also raises the possibility that a quantitative map could complement pharmacogenomics

OPEN
ACCESS
CellPress

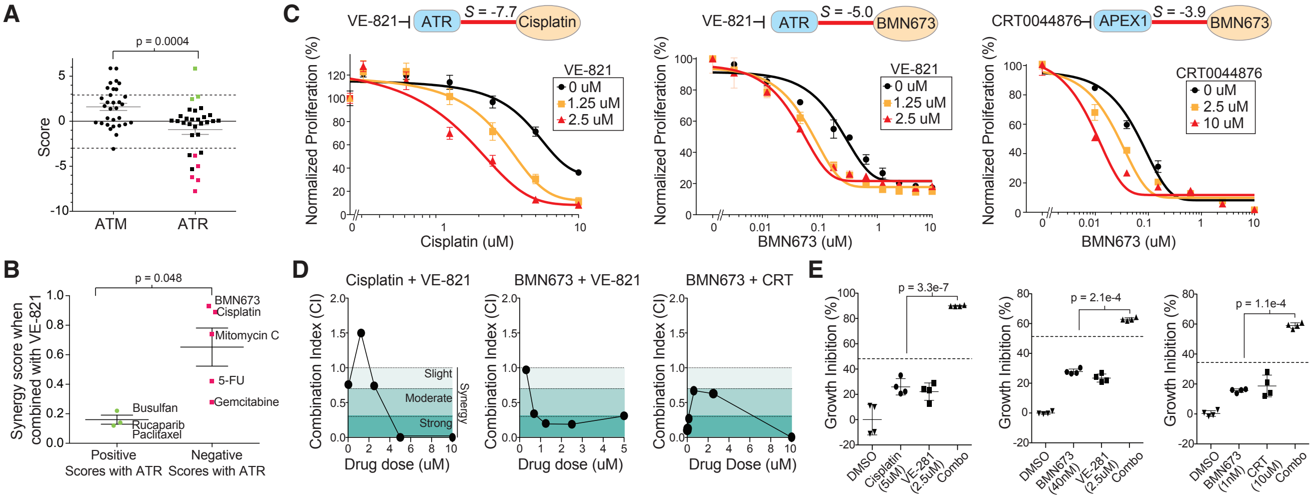

Figure 3. Prediction of Drug Synergies Using the Chemical-Genetic Interaction Map

(A) Comparison of chemical interaction scores for ATM and ATR knockdown. Positive (green) and negative (red) drugs were selected for combination testing.

(B) Synergy scores between the ATR inhibitor VE-821 and selected drugs.

(C) Relative proliferation of MCF10A cells treated with cisplatin or BMN673 alone or in combination with the indicated dose of VE-821 or CRT0044876 (APEX1 inhibitor) for 72 hr. Drug combinations are normalized to the indicated dose of VE-821 or CRT0044876 alone.

(D) Combination index (CI) values for combinations. Shaded regions represent synergistic CI values indicating strong synergy (<0.3), moderate synergy (0.3–0.7), or slight synergy (0.7–1). Drug concentrations are at a fixed ratio of 1:1 μM, except for CRT0044876 (CRT), which is fixed at 10 μM.

(E) Percent growth inhibition of MCF10A cells treated with DMSO, a single drug, or drug combinations for 72 hr at the indicated dose. Dotted line represents expected growth inhibition based on drug additivity. Significance is based on comparison of the observed growth inhibition to this expected value.

Error bars indicate SD except in (A) and (B), where they indicate SEM.

efforts using large panels of cancer cell lines (Barretina et al., 2012; Basu et al., 2013; Garnett et al., 2012). While previous studies have used supervised machine-learning approaches to identify molecular correlates of drug sensitivity across cell lines, we hypothesized that the relationships identified by gene knockdown constitute a more direct and causal readout of gene function that could enhance biomarker identification. Comparison of 11 drugs in common between our study and the Cancer Therapeutics Response Portal (CTRP) revealed a strong degree of overlap between interactions identified in the chemical-genetic interaction map and genes whose response was significantly correlated with the drug response (Figure 2B) (Basu et al., 2013). Furthermore, this degree of overlap was highly related to the score threshold used with 21.5% of interactions overlapping at a cutoff of 3 ($p = 2.9e-3$) and nearly 60% overlapping at a cutoff of 8 ($p = 3.1e-5$), reflecting the quantitative nature of the dataset (Figure 2B). One example of an interaction recapitulated in cell lines was ARID1A and etoposide, with a score of 8.15. This interaction was observed across 496 cancer cell lines where ARID1A expression was strongly linked with etoposide sensitivity ($r = -0.297$, $p = 1.5e-11$) and low ARID1A expression was highly predictive of resistance to etoposide (94.8% precision; Figures S2A and S2B). This interaction was present in the majority of the 22 tumor lineages analyzed and strongest in sarcoma lines ($r = -0.9$; Figure S2C). Etoposide impairs replication and causes DNA double-strand breaks by locking topoisomerase II onto DNA (Pommier et al., 2010). Since ARID1A facilitates the binding of topoisomerase II onto DNA (Dykhuizen et al., 2013), ARID1A loss could contribute to resistance by impairing the cytotoxic effect of etoposide. Therefore, genetic interaction information complements cell-line screening efforts and may be used to generate mechanistic hypotheses that bridge between correlation and causation.

The significance and quantitative nature of the overlap between our map and expression-based correlates of drug sensitivity found in cancer cell lines prompted us to explore whether this map could be used to systematically predict cancer cell line sensitivity in an unsupervised fashion. For each drug, we used the relative expression of each of the genes in its network to derive a drug response prediction for every cell line (Experimental Procedures). We evaluated this method using a sliding cutoff to define the specific network for each drug and found that more stringent networks provided increased power to predict drug sensitivity, with nearly 60%–70% of drugs predicted accurately at a cutoff between 5 and 6 (Figure 2C). At a cutoff of 5, predictions were significant for 7 out of the 11 drugs (Figure 2D). Analysis of the genes that were most informative in making correct predictions in these cases revealed genes involved in drug sensitivity and resistance. Knockdown of EIF4A1 caused resistance to methotrexate ($S = 6.5$), and in cell lines, EIF4A1 expression is positively correlated with methotrexate sensitivity across 645 cell lines ($r = 0.25$, $p = 1.9e-8$), consistent with the network prediction. Alternatively, SNX24 knockdown was synthetic lethal with paclitaxel ($S = -6.8$), and SNX24 expression was negatively correlated with drug sensitivity ($r = -0.14$, $p = 0.0026$). Thus, computational analysis of chemical-genetic interaction maps can be used to complement cancer cell line screens and may be able to produce biomarkers that bridge correlation with causation.

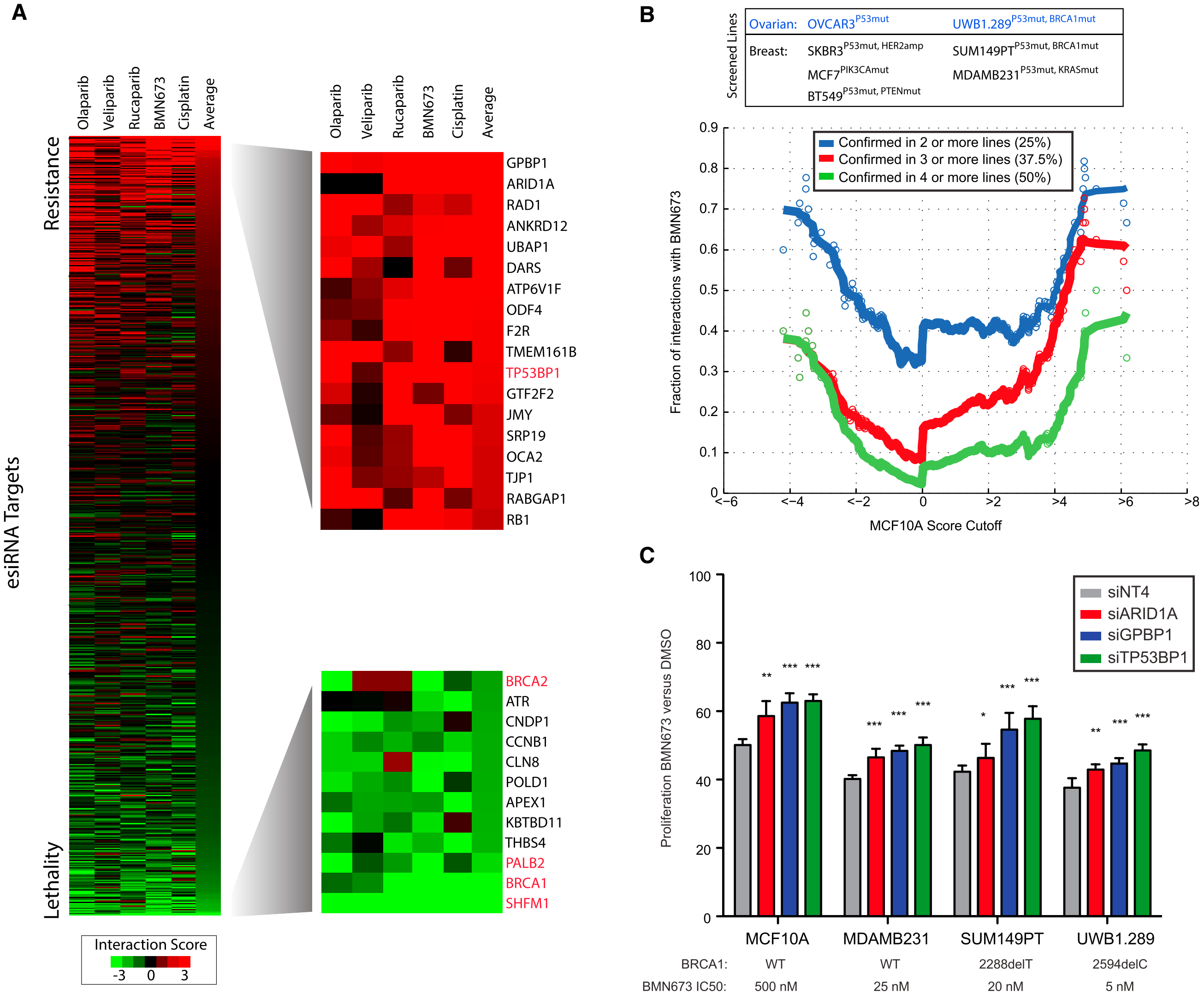

Figure 4. Assessment of Genetic Interactions with PARP Inhibitors and Cisplatin

(A) Interaction profiles of four PARP inhibitors and cisplatin sorted based on average across all 5 drugs. Known factors associated with resistance and sensitivity are indicated in red.

(B) Assessment of the preservation of interactions between MCF10A cells and seven cancer cell lines measured through a rescreen of BMN673 chemical-genetic interactions. A genetic interaction is considered preserved if it is significant ($p < 0.01$), with the same direction in one or more lines. Each point represents the cumulative rate of preservation for all interactions scoring past a particular cutoff. Solid lines indicate sliding averages.

(C) Confirmation of resistance interactions using independent synthetic siRNA gene knockdown in cell lines. Knockdown samples were treated with an approximate IC_{50} dose of BMN673 and normalized treatment with DMSO. siTP53BP1 was used as a positive control for resistance, and siNT4 is the non-targeting control.

Data indicate mean ± SD. *p < 0.05; **p < 0.005; ***p < 0.0005, by two tailed Student’s t test.

See also Table S4.

Prediction of Drug Synergies Using the Chemical-Genetic Interaction Map

There has been considerable interest in the development of targeted therapies that inhibit DNA-repair machinery to be used in combination with agents that generate specific types of DNA damage (Gavande et al., 2016). We observed that loss of the DNA-damage signaling kinase ATR induced sensitivity to cross-linking agents and inhibitors of DNA replication in the consensus map (Figure 2A). This was in contrast to its closely related paralog kinase ATM, which was not linked to the response to these drugs (Figure 2A). We hypothesized that the synthetic lethal interactions observed with ATR knockdown could be phenocopied with a small-molecule inhibitor of ATR and used to prioritize synergistic drug combinations. We tested the combined effects of the ATR inhibitor VE-821 with five drugs that were synthetic lethal with ATR knockdown and three drugs that were not (Figure 3A). Using a matrix screening approach, we measured the effects of each drug on proliferation and determined a

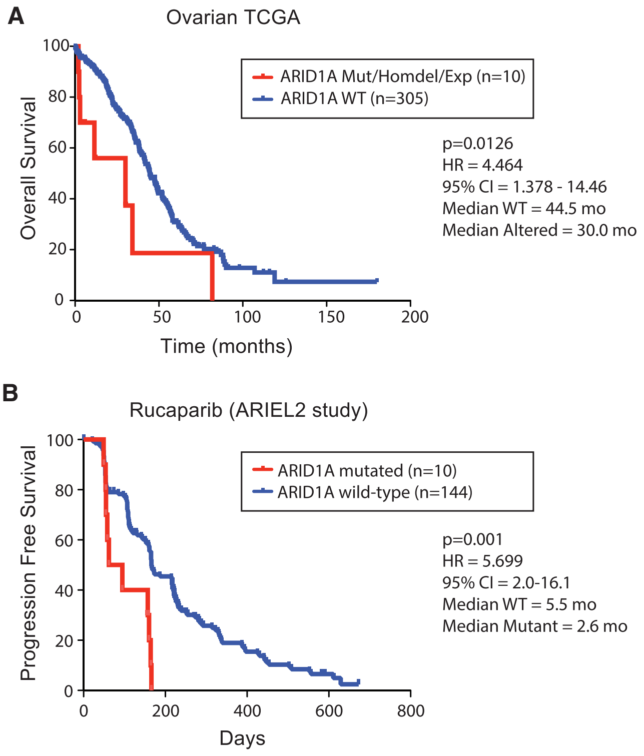

Figure 5. ARID1A Loss Associates with Platinum and PARP Inhibitor Resistance in Patient Cohorts

(A) Impact of ARID1A loss, determined via mutation, homozygous deletion, or loss of expression, and survival in TCGA serous ovarian cancers.

(B) Progression-free survival of relapsed, platinum-sensitive, high-grade ovarian carcinomas in a clinical trial of rucaparib, stratified based on ARID1A mutation status.

The p values are based on log-rank test. HR, hazard ratio; CI, confidence interval of survival.

See also Figure S3 and Table S5.

synergy score reflecting the degree of drug synergy based on the Loewe excess model (Lehár et al., 2009). Drugs that were synthetic lethal with ATR inhibition were more synergistic than those that were not predicted to be ($p = 0.048$, Figure 3B). In particular, we found that the addition of VE-821 could sensitize MCF10A cells to cisplatin (ATR, $S = -7.7$) and BMN673 (ATR, $S = -5.0$) (Figure 3C), with a combination index (CI) that was below 1 for most dose combinations, indicative of true synergy (Figure 3D) (Chou, 2010). Analysis at doses where synergy was apparent revealed that the combination had a greater than additive impact on growth inhibition (Figure 3E). We also identified other druggable nodes in the chemical-genetic interaction map and tested whether resulting combinations had evidence of drug synergy. The map identified an interaction between the loss of the base excision repair protein APEX1 and PARP inhibition that was the lowest with BMN673 ($S = -3.9$) (Figure 2A). We tested for potential synergy between BMN673 and a small-molecule inhibitor of APEX1, CRT0044876 (Madhusudan et al., 2005). CRT0044876 displayed a dose-dependent ability to sensitize MCF10A cells to BMN673 (Figure 3C) and showed evidence of synergy via CI calculation as well as a greater than additive response (Figures 3D and 3E). Therefore, the chemical-genetic interaction map can be used to prioritize drug combinations, which may increase the efficacy of chemotherapeutics.

Prediction of Factors Mediating Resistance to PARP Inhibition

We next evaluated the map as a systematic resource to predict molecules involved in DNA repair and associated mechanisms of resistance to chemotherapy. We focused on PARP inhibitors olaparib, veliparib, rucaparib, and BMN673, as well as cisplatin, since this class was highly covered in the map, has similar mechanisms of action, and generates DNA damage that depends on repair via HR (Figure 1E). As controls, BRCA1, BRCA2, PALB2, and SHFM1 knockdown was synthetic lethal with these agents, and loss of TP53BP1 was associated with resistance (Figure 4A). An important consideration in interpreting genetic interaction data from a single cell line is the degree to which such interactions are maintained in other cellular contexts (Ashworth et al., 2011). To assess whether interactions were maintained, we identified chemical-genetic interactions using the same experimental approach with BMN673 in a total of 7 additional lines, including four breast cancer lines and three ovarian cancer lines. After scoring esiRNAs for their ability to induce resistance or sensitivity to BMN673 in each of these lines (defined based on a cutoff of $p < 0.01$), we found that the chemical-genetic interaction score was highly predictive of whether a particular interaction was preserved in other cell lines (Table S4). For example, interactions with BMN673 that had a score >5 in MCF10A cells were 40% likely to be validated in 4 or more cell lines (half of all 8 lines tested) and 70% likely to be validated in at least 2 other lines (Figure 4B). This trend was also evident for negative interactions, where an S score < -4 had a 40% chance of being scored as a synthetic lethal in half the cell lines tested. We note that no interaction was scored as synthetic lethal in all cell lines. Since two of the tested lines were *BRCA1* mutant (SUM149PT, UWB1.289), a likely reason for this is that factors whose loss leads to PARP sensitivity in HR-competent MCF10A cells may not be relevant in *BRCA1* mutant cells that are already HR deficient. Therefore, these data suggest that interaction strength in MCF10A cells can be used to predict genetic interactions in other cell lines, highlighting the quantitative nature of this map.

We next sought to validate the top two hits producing resistance, GPBP1 and ARID1A, in additional models. Using independent siRNAs, we confirmed that loss of either factor caused resistance to BMN673 in MCF10A, MDAMB231, SUM149PT, and UWB1.289 cells at levels comparable to that for loss of TP53BP1 as a positive control for PARP inhibitor resistance (Figure 4C). ARID1A loss often occurs through somatic mutation and has been previously linked to the regulation of DNA-repair processes (Dykhuizen et al., 2013; Shen et al., 2015). We confirmed this result in engineered ARID1A$^{-/-}$ MCF10A cells, which were resistant to BMN673 in comparison to parental MCF10A cells (Figures S3A and S3B). We next searched for clinical evidence that ARID1A loss contributes to resistance to PARP or platinum-containing chemotherapy. In support, we found that ARID1A loss was linked to poor outcome in TCGA high-grade serous ovarian cancers (TCGA HGSOCs) receiving platinum as the standard of care ($p = 0.01$; Figure 5A; Table S5) (Cancer Genome Atlas

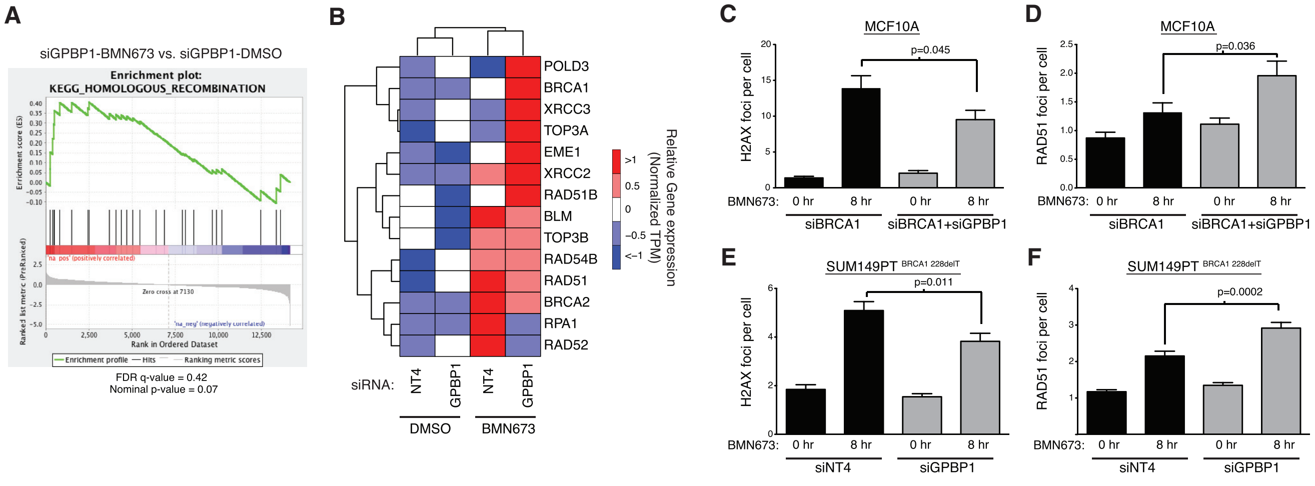

Figure 6. GPBP1 Loss Causes Upregulation of the Homologous Recombination Pathway

(A) Gene set enrichment analysis of homologous recombination pathway genes using RNA-seq data from GPBP1-knockdown MCF10A cells treated with 0.5 μM BMN673 or DMSO for 24 hr.

(B) Heatmap representation of expression of HR pathway genes differentially expressed in the presence of BMN673. TPM, transcripts per kilobase million.

(C and D) Quantification of gamma-H2AX foci (C) and RAD51 recruitment (D) after treatment with 500 nM of BMN673 in the presence of the indicated gene knockdowns in MCF10A cells.

(E and F) Quantification of gamma-H2AX foci (E) and RAD51 recruitment (F) after treatment with 50 nM of BMN673 in the presence of the indicated gene knockdowns in SUM149PT cells. NT4 was the non-targeting control.

Error bars indicate SEM.

See also Figure S4.

Research Network, 2011). To test whether this observation extended to PARP inhibitors, we analyzed samples from HGSOC patients treated with rucaparib in a phase II clinical trial (ClinicalTrials.gov number NCT01891344) (Swisher et al., 2017). We did not identify any patients with concurrent *BRCA1* and *ARID1A* mutations and, therefore, focused our analysis on a cohort of 154 patients without mutations in HR pathway genes and identified 10 that had mutations in ARID1A. The progression-free survival (PFS) for these 10 *ARID1A* mutant cases was significantly lower than for the rest of this cohort ($p = 0.003$; Figure 5B). All ARID1A mutant cases were HGSOCs, confirmed by histological analysis (Table S5). These clinical data show that PARP inhibitors provide no clinical benefit in ARID1A-mutated, high-grade serous or endometrioid ovarian cancer and warrants further investigation.

GPBP1 Loss Causes PARP and Platinum Resistance by Regulating the Expression of Factors Involved in HR

We next investigated the top candidate in our categorical analysis, GPBP1, a transcription factor of unknown function. GPBP1 lies on chromosome 5q11, a region focally deleted in approximately 5% of TCGA HGSOCs and 4% of TCGA breast cancers. To determine whether GPBP1 plays a role in the transcriptional response to DNA damage, we performed RNA-sequencing (RNA-seq) analysis of control and GPBP1-knockdown MCF10A cells treated with or without BMN673. qRT-PCR of GPBP1-knockdown cells confirmed 90% knockdown at the mRNA level in this experiment (Figure S4A). In response to 24-hr BMN673 treatment, GPBP1 knockdown caused the upregulation of factors involved in HR based on gene set enrichment analysis (GSEA) (Subramanian et al., 2005) (Figure 6A), indicating a potential compensatory mechanism to facilitate repair of lesions incurred by PARP inhibition. In contrast to control cells, GPBP1 knockdown resulted in the upregulation of distinct and canonical HR factors such as BRCA1 and RAD51B in response to BMN673 (Figure 6B).

We next asked whethr this transcriptional response was sufficient to enhance the repair of double-strand breaks incurred by PARP inhibition and whether this occurred via HR. This hypothesis was particularly intriguing, since GPBP1 knockdown caused resistance to BMN673 in *BRCA1* mutant cancer cell lines, suggesting that GPBP1 loss may bypass the requirement of BRCA1 for HR (Figure 4C). To test this hypothesis, we established an HR-deficient and PARP-inhibitor-sensitive MCF10A model by BRCA1 knockdown, and in this model, knockdown of BRCA1 and GPBP1 together led to a significant rescue of BMN673 sensitivity (Figure S4B). Using immunofluorescence, we found that generation of γH2AX foci after BMN673 treatment was reduced in BRCA1+GPBP1 versus BRCA1 knockdown cells ($p = 0.045$), indicating that GPBP1 loss led to a reduction in the number of DNA double-strand breaks formed after PARP inhibitor treatment (Figure 6C). To determine whether this reduction in double-strand breaks was due to heightened HR repair capacity, we examined the recruitment of the strand-exchange protein RAD51 to damaged chromatin, a mark of commitment to double-strand break repair using HR. We found that the recruitment of RAD51 was increased in BRCA1+GPBP1 versus BRCA1 knockdown cells after PARP inhibition, indicating that GPBP1 loss led to an increase in double-strand break repair through HR ($p = 0.036$; Figure 6D). We confirmed these findings in

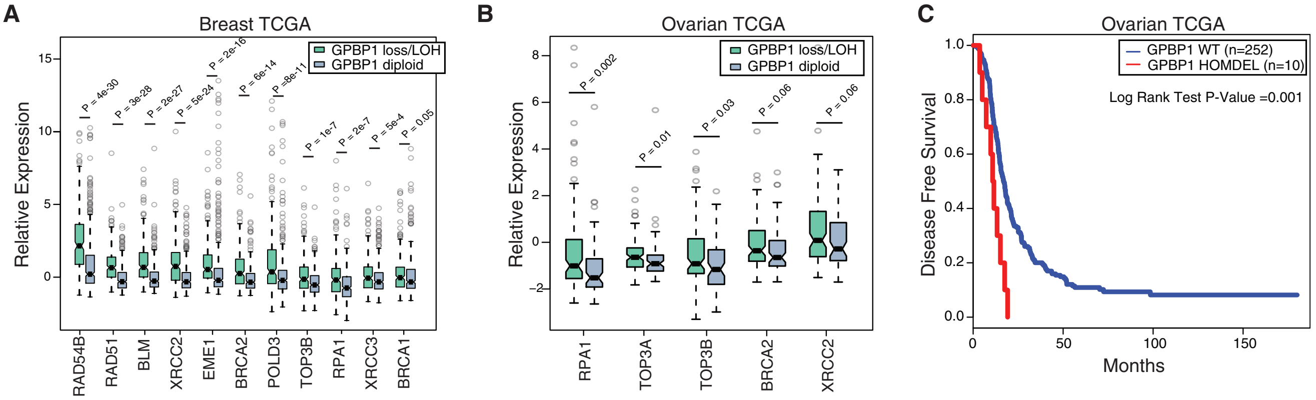

Figure 7. Cancers with GPBP1 Loss Display Heightened Expression of HR Genes and Resist Platinum Treatment in Ovarian Cancer

(A) Comparison of gene expression levels of homologous recombination pathway genes in TCGA breast cancers among tumors with GPBP1 homozygous/heterozygous loss versus diploid copy number variation (CNV) status.

(B) Comparison of gene expression levels of homologous recombination pathway genes in ovarian cancer patients from the TCGA HGSOC dataset with GPBP1 homozygous/heterozygous loss versus diploid CNV status. The p values were calculated by non-parametric Mann-Whitney-Wilcoxon test.

(C) The Kaplan-Meier disease-free survival (DFS) analysis of patients with TCGA HGSOC, with samples with deletion in GPBP1. Boxes represent the interquartile range, and whiskers indicate 1.5 times the interquartile range.

BRCA1 mutant SUM149PT cells, where GPBP1 knockdown also led to a significant reduction in H2AX foci and increase in RAD51 foci after BMN673 treatment (Figures 6E and 6F), indicating that GPBP1 loss can also restore HR in cases when *BRCA1* is mutated. These results indicate that GPBP1 loss contributes to increased double-strand break repair by HR as a mechanism of PARP inhibitor resistance.

Drawing from our RNA-seq analysis, we next asked whether the expression of HR factors was also elevated in human cancer samples harboring GPBP1 loss and whether it might contribute to drug resistance. There was a strong concordance between genes upregulated upon GPBP1 knockdown and genes whose expression level was higher in breast cancers with GPBP1 loss (Figure 7A). Further analysis of samples with GPBP1 loss in TCGA ovarian cancer samples also reflected the increased expression of a number of the same HR factors, indicating a similar function of GPBP1 in these two disease types (Figure 7B). We next asked whether this enhancement in HR gene expression upon GPBP1 loss resulted in drug resistance in ovarian cancer patients treated with platinum-containing therapy. In the TCGA ovarian cohort, survival analysis indicated that GPBP1 loss was associated with poor outcome and resistance to platinum therapy ($p = 0.001$, via log-rank test; Figure 7C). Therefore, GPBP1 loss contributes to platinum resistance in ovarian cancer through the increased expression of genes involved in HR.

DISCUSSION

We present a quantitative map to link the efficacy of chemotherapeutics to tumor genetics that can serve as a platform for the functional and therapeutic translation of tumor genomes. In contrast to most standard genetic screens, this approach provides a quantitative readout that approximates genetic interaction strength and allows for the comparison of responses across many drugs. This map is able to recapture many known synthetic lethal interactions, and future work may expand on this map beyond the set of genes screened here, as well as using complementary technologies such as CRISPR/Cas9 to evaluate the impact of gene knockout versus knockdown as well as gene activation. To aid in integration of these data with ongoing efforts to systematize cancer-related screens, data from this network have been deposited into the Cancer Target Discovery and Development (CTD^2) Dashboard (https://ctd2-dashboard.nci.nih.gov/).

Using insights derived from the chemical-genetic interaction map, we highlight several vignettes describing how it can be used to aid in the development of cancer therapeutics. The map was able to identify drugs with similar mechanisms of action and highlights the mechanistic commonalities between PARP inhibitors and DNA cross-linking agents that contribute to synthetic lethality with loss of HR pathway genes. The map identified interactions that could be recapitulated with small-molecule inhibitors of ATR and APEX1, revealing synergistic drug combinations. As a means to highlight the quantitative nature of this resource for more systematic discovery, we show that computational analysis of this map can be used to predict the sensitivity of tumor cells to chemotherapies. As many large-scale efforts to screen cancer-cell-line panels with small molecules are ongoing (Barretina et al., 2012; Basu et al., 2013; Garnett et al., 2012), our analysis suggests that loss of many of the factors identified in this map is linked with drug sensitivity in a predictive way. Since current approaches do not use this functional information, the map may provide a platform for enhancing methods to predict drug responses from baseline genomic profiles by bridging correlation with causation (Costello et al., 2014). Future computational approaches to integrate cell-line screen with chemical-genetic interaction maps could aid in these more established drug and biomarker development approaches.

We demonstrate several ways to enhance the reliability and utility of this map. First, we show that related drugs have similar genetic interaction profiles and that this property can be used to

identify modifiers of therapeutic responses that are not specific to a single compound. As specific drugs may have unique off-targets, such as the case for PARP inhibitors (Knezevic et al., 2016), analyzing related drugs together may identify genetic interactions linked to their core mechanism of action. Second, the plasticity in genetic networks has been an impediment to the identification of genetic interactions that are cell type independent (i.e., "hard" versus "soft" interactions) (Ashworth et al., 2011). Rescreening in multiple cancer cell lines showed that the strength of genetic interaction was proportional to the likelihood of interaction being conserved in other cell lines. Therefore our data indicate that the quantitative nature of genetic interaction maps could be used to distinguish between interactions that are more globally preserved versus those more specific to the cell line tested.

Based on our categorical analysis, we identified that ARID1A and GPBP1 loss causes PARP inhibitor resistance. Low ARID1A expression has been linked with poor outcome and platinum resistance in HGSOC (Yokoyama et al., 2014) and clear-cell ovarian cancers (Itamochi et al., 2015; Katagiri et al., 2012). However, the functional role of ARID1A on DNA repair is unclear, with conflicting reports of its role in HR (Adamson et al., 2012; Shen et al., 2015). Together, these data warrant a more complete interrogation of the role of ARID1A on PARP inhibitor resistance. The strongest resistance interaction with PARP inhibitors and cisplatin was GPBP1, which, as we show, is involved in the transcriptional regulation of genes involved in HR. Another transcriptional regulator, CDK12, has been shown to modulate PARP inhibitor sensitivity in a similar manner (Bajrami et al., 2014; Johnson et al., 2016). Future studies may seek to identify the potential interplay between the targets of CDK12 and GPBP1. Since we observed that GPBP1 loss is also linked to chemoresistance and poor clinical outcome, these data warrant a more complete interrogation of the function and role of GPBP1. Because GPBP1 loss was not assayed in our rucaparib clinical trial cohort, future work could determine its clinical association with PARP inhibitor resistance. This work highlights the utility of a systematic chemical-genetic interaction map as a resource for the identification of clinically relevant biomarkers of drug susceptibility, as well as a foundation for integration with other cancer datasets to enhance drug and biomarker development.

EXPERIMENTAL PROCEDURES

Cell Culture

MCF10A cells were maintained in DMEM/F12 medium supplemented with 5% horse serum, epidermal growth factor (20 ng/mL), insulin (10 μg/mL), hydrocortisone (0.5 mg/mL), cholera toxin (100 ng/mL), and penicillin/streptomycin. All cell lines were obtained from the ATCC and cultured according to listed protocols, except for ARID1A$^{-/-}$ MCF10A cells, which were obtained from Horizon Discovery (Cambridge, UK) and maintained in the MCF10A media described earlier. Rucaparib was provided by Clovis Oncology; other drugs were purchased from Selleckchem.

Measurement of Chemical-Genetic Interactions

MCF10A cells were reverse transfected in 384-well plates (1,000 cells per well) using 5 ng of esiRNA (Sigma) with RNAiMax (0.05 μL per well) as a transfection reagent in quadruplicate. Cells were transfected for 24 hr, and then the entire plate was treated with one drug at a half maximal inhibitory concentration (IC_{50}) concentration or DMSO for 72 hr, after which cells were stained with Hoescht 33342 and counted using a Thermo CellInsight high-content microscope.

After drug or DMSO treatment, each plate was median centered to 2,000 cells per well to normalize relative proliferation rates. Plates had a minimum internal correlation across the 4 replicate wells of 0.7. Each well in the drug-treated plate was then compared to the same well in the DMSO-treated plate. We observed an overall linear relationship between drug and DMSO plates, indicating that most esiRNAs have no effect on drug sensitivity. Next, the set of 4 normalized replicate values in the DMSO plate was compared to the same in the drug plate, and both the fold change in cell number and the p value of significance of this difference in medians were calculated using a modified t test. The *S* score of genetic interaction is defined by the negative $\log_{10}$ of the t test p value and signed with either positive (gene loss drives resistance to drug) or negative (gene loss drives sensitivity to drug) values. FDR was calculated based on the percentage of negative-control knockdowns (GFP) whose score exceeded a given threshold. The described protocol is available in MATLAB, and code and raw data to recreate the dataset are available at https://github.com/BandyopadhyayLab/.

DATA AND SOFTWARE AVAILABILITY

The accession number for the sequencing data reported in this paper is GEO: GSE101904.

SUPPLEMENTAL INFORMATION

Supplemental Information includes Supplemental Experimental Procedures, four figures, and five tables and can be found with this article online at https://doi.org/10.1016/j.celrep.2018.03.093.

ACKNOWLEDGMENTS

This work was funded by NCI grant U01CA217882, NIGMS grant R01GM107671, the UCSF Program in Breakthrough Biomedical Research (PBBR), the Prospect Creek Foundation, and a UCSF Beast Oncology SPORE development award to S.B. We thank members of the Bandyopadhyay lab and Sophia Pfister for helpful comments and critical review of the manuscript and Hayley Donnella for technical assistance.

AUTHOR CONTRIBUTIONS

H.-M.H., S.B., and T.C.H. conceived the study. H.-M.H., X.Z., S.K., K.N.S., L.R., A.B., K.K.L., and S.B. generated data and participated in computational analyses. A.D.S., M.R., T.C.H., and S.B. administered the project. S.B. supervised the study. All authors wrote and approved the manuscript.

DECLARATION OF INTERESTS

L.R., K.K.L., A.D.S., M.R. and T.C.H. are employees of Clovis Oncology. S.B. is a consultant for Ideaya Biosciences.

Received: July 24, 2017
Revised: February 7, 2018
Accepted: March 20, 2018
Published: April 17, 2018

REFERENCES

Adamson, B., Smogorzewska, A., Sigoillot, F.D., King, R.W., and Elledge, S.J. (2012). A genome-wide homologous recombination screen identifies the RNA-binding protein RBMX as a component of the DNA-damage response. Nat. Cell Biol. *14*, 318–328.

Ashworth, A., Lord, C.J., and Reis-Filho, J.S. (2011). Genetic interactions in cancer progression and treatment. Cell *145*, 30–38.

Bajrami, I., Frankum, J.R., Konde, A., Miller, R.E., Rehman, F.L., Brough, R., Campbell, J., Sims, D., Rafiq, R., Hooper, S., et al. (2014). Genome-wide

profiling of genetic synthetic lethality identifies CDK12 as a novel determinant of PARP1/2 inhibitor sensitivity. Cancer Res. *74*, 287–297.

Barcenas, C.H., Niu, J., Zhang, N., Zhang, Y., Buchholz, T.A., Elting, L.S., Hortobagyi, G.N., Smith, B.D., and Giordano, S.H. (2014). Risk of hospitalization according to chemotherapy regimen in early-stage breast cancer. J. Clin. Oncol. *32*, 2010–2017.

Barretina, J., Caponigro, G., Stransky, N., Venkatesan, K., Margolin, A.A., Kim, S., Wilson, C.J., Lehár, J., Kryukov, G.V., Sonkin, D., et al. (2012). The Cancer Cell Line Encyclopedia enables predictive modelling of anticancer drug sensitivity. Nature *483*, 603–607.

Basu, A., Bodycombe, N.E., Cheah, J.H., Price, E.V., Liu, K., Schaefer, G.I., Ebright, R.Y., Stewart, M.L., Ito, D., Wang, S., et al. (2013). An interactive resource to identify cancer genetic and lineage dependencies targeted by small molecules. Cell *154*, 1151–1161.

Bouwman, P., Aly, A., Escandell, J.M., Pieterse, M., Bartkova, J., van der Gulden, H., Hiddingh, S., Thanasoula, M., Kulkarni, A., Yang, Q., et al. (2010). 53BP1 loss rescues BRCA1 deficiency and is associated with triple-negative and BRCA-mutated breast cancers. Nat. Struct. Mol. Biol. *17*, 688–695.

Bryant, H.E., Schultz, N., Thomas, H.D., Parker, K.M., Flower, D., Lopez, E., Kyle, S., Meuth, M., Curtin, N.J., and Helleday, T. (2005). Specific killing of BRCA2-deficient tumours with inhibitors of poly(ADP-ribose) polymerase. Nature *434*, 913–917.

Buisson, R., Dion-Côté, A.M., Coulombe, Y., Launay, H., Cai, H., Stasiak, A.Z., Stasiak, A., Xia, B., and Masson, J.Y. (2010). Cooperation of breast cancer proteins PALB2 and piccolo BRCA2 in stimulating homologous recombination. Nat. Struct. Mol. Biol. *17*, 1247–1254.

Bunting, S.F., Callén, E., Wong, N., Chen, H.T., Polato, F., Gunn, A., Bothmer, A., Feldhahn, N., Fernandez-Capetillo, O., Cao, L., et al. (2010). 53BP1 inhibits homologous recombination in Brca1-deficient cells by blocking resection of DNA breaks. Cell *141*, 243–254.

Byrski, T., Dent, R., Blecharz, P., Foszczynska-Kloda, M., Gronwald, J., Huzarski, T., Cybulski, C., Marczyk, E., Chrzan, R., Eisen, A., et al. (2012). Results of a phase II open-label, non-randomized trial of cisplatin chemotherapy in patients with BRCA1-positive metastatic breast cancer. Breast Cancer Res. *14*, R110.

Cancer Genome Atlas Network (2012). Comprehensive molecular portraits of human breast tumours. Nature *490*, 61–70.

Cancer Genome Atlas Research Network (2011). Integrated genomic analyses of ovarian carcinoma. Nature *474*, 609–615.

Chapman, J.R., Sossick, A.J., Boulton, S.J., and Jackson, S.P. (2012). BRCA1-associated exclusion of 53BP1 from DNA damage sites underlies temporal control of DNA repair. J. Cell Sci. *125*, 3529–3534.

Cheung-Ong, K., Giaever, G., and Nislow, C. (2013). DNA-damaging agents in cancer chemotherapy: serendipity and chemical biology. Chem. Biol. *20*, 648–659.

Chou, T.C. (2010). Drug combination studies and their synergy quantification using the Chou-Talalay method. Cancer Res. *70*, 440–446.

Costello, J.C., Heiser, L.M., Georgii, E., Gönen, M., Menden, M.P., Wang, N.J., Bansal, M., Ammad-ud-din, M., Hintsanen, P., Khan, S.A., et al.; NCI DREAM Community (2014). A community effort to assess and improve drug sensitivity prediction algorithms. Nat. Biotechnol. *32*, 1202–1212.

Curtis, C., Shah, S.P., Chin, S.F., Turashvili, G., Rueda, O.M., Dunning, M.J., Speed, D., Lynch, A.G., Samarajiwa, S., Yuan, Y., et al.; METABRIC Group (2012). The genomic and transcriptomic architecture of 2,000 breast tumours reveals novel subgroups. Nature *486*, 346–352.

Debnath, J., Mills, K.R., Collins, N.L., Reginato, M.J., Muthuswamy, S.K., and Brugge, J.S. (2002). The role of apoptosis in creating and maintaining luminal space within normal and oncogene-expressing mammary acini. Cell *111*, 29–40.

Dykhuizen, E.C., Hargreaves, D.C., Miller, E.L., Cui, K., Korshunov, A., Kool, M., Pfister, S., Cho, Y.J., Zhao, K., and Crabtree, G.R. (2013). BAF complexes facilitate decatenation of DNA by topoisomerase IIα. Nature *497*, 624–627.

Early Breast Cancer Trialists' Collaborative Group (EBCTCG) (2005). Effects of chemotherapy and hormonal therapy for early breast cancer on recurrence and 15-year survival: an overview of the randomised trials. Lancet *365*, 1687–1717.

Farmer, H., McCabe, N., Lord, C.J., Tutt, A.N., Johnson, D.A., Richardson, T.B., Santarosa, M., Dillon, K.J., Hickson, I., Knights, C., et al. (2005). Targeting the DNA repair defect in BRCA mutant cells as a therapeutic strategy. Nature *434*, 917–921.

Garnett, M.J., Edelman, E.J., Heidorn, S.J., Greenman, C.D., Dastur, A., Lau, K.W., Greninger, P., Thompson, I.R., Luo, X., Soares, J., et al. (2012). Systematic identification of genomic markers of drug sensitivity in cancer cells. Nature *483*, 570–575.

Gavande, N.S., VanderVere-Carozza, P.S., Hinshaw, H.D., Jalal, S.I., Sears, C.R., Pawelczak, K.S., and Turchi, J.J. (2016). DNA repair targeted therapy: The past or future of cancer treatment? Pharmacol. Ther. *160*, 65–83.

Helleday, T. (2011). The underlying mechanism for the PARP and BRCA synthetic lethality: clearing up the misunderstandings. Mol. Oncol. *5*, 387–393.

Itamochi, H., Oumi, N., Oishi, T., Shoji, T., Fujiwara, H., Sugiyama, T., Suzuki, M., Kigawa, J., and Harada, T. (2015). Loss of ARID1A expression is associated with poor prognosis in patients with stage I/II clear cell carcinoma of the ovary. Int. J. Clin. Oncol. *20*, 967–973.

Johnson, S.F., Cruz, C., Greifenberg, A.K., Dust, S., Stover, D.G., Chi, D., Primack, B., Cao, S., Bernhardy, A.J., Coulson, R., et al. (2016). CDK12 inhibition reverses de novo and acquired PARP inhibitor resistance in BRCA wild-type and mutated models of triple-negative breast cancer. Cell Rep. *17*, 2367–2381.

Katagiri, A., Nakayama, K., Rahman, M.T., Rahman, M., Katagiri, H., Nakayama, N., Ishikawa, M., Ishibashi, T., Iida, K., Kobayashi, H., et al. (2012). Loss of ARID1A expression is related to shorter progression-free survival and chemoresistance in ovarian clear cell carcinoma. Mod. Pathol. *25*, 282–288.

Kittler, R., Surendranath, V., Heninger, A.K., Slabicki, M., Theis, M., Putz, G., Franke, K., Caldarelli, A., Grabner, H., Kozak, K., et al. (2007). Genome-wide resources of endoribonuclease-prepared short interfering RNAs for specific loss-of-function studies. Nat. Methods *4*, 337–344.

Knezevic, C.E., Wright, G., Rix, L.L.R., Kim, W., Kuenzi, B.M., Luo, Y., Watters, J.M., Koomen, J.M., Haura, E.B., Monteiro, A.N., et al. (2016). Proteome-wide profiling of clinical PARP inhibitors reveals compound-specific secondary targets. Cell Chem. Biol. *23*, 1490–1503.

Lehár, J., Krueger, A.S., Avery, W., Heilbut, A.M., Johansen, L.M., Price, E.R., Rickles, R.J., Short, G.F., 3rd, Staunton, J.E., Jin, X., et al. (2009). Synergistic drug combinations tend to improve therapeutically relevant selectivity. Nat. Biotechnol. *27*, 659–666.

Liu, X., Shi, Y., Maag, D.X., Palma, J.P., Patterson, M.J., Ellis, P.A., Surber, B.W., Ready, D.B., Soni, N.B., Ladror, U.S., et al. (2012). Iniparib nonselectively modifies cysteine-containing proteins in tumor cells and is not a bona fide PARP inhibitor. Clin. Cancer Res. *18*, 510–523.

Lord, C.J., and Ashworth, A. (2013). Mechanisms of resistance to therapies targeting BRCA-mutant cancers. Nat. Med. *19*, 1381–1388.

Madhusudan, S., Smart, F., Shrimpton, P., Parsons, J.L., Gardiner, L., Houlbrook, S., Talbot, D.C., Hammonds, T., Freemont, P.A., Sternberg, M.J., et al. (2005). Isolation of a small molecule inhibitor of DNA base excision repair. Nucleic Acids Res. *33*, 4711–4724.

Martins, M.M., Zhou, A.Y., Corella, A., Horiuchi, D., Yau, C., Rakshandehroo, T., Gordan, J.D., Levin, R.S., Johnson, J., Jascur, J., et al. (2015). Linking tumor mutations to drug responses via a quantitative chemical-genetic interaction map. Cancer Discov. *5*, 154–167.

McCabe, N., Turner, N.C., Lord, C.J., Kluzek, K., Bialkowska, A., Swift, S., Giavara, S., O'Connor, M.J., Tutt, A.N., Zdzienicka, M.Z., et al. (2006). Deficiency in the repair of DNA damage by homologous recombination and sensitivity to poly(ADP-ribose) polymerase inhibition. Cancer Res. *66*, 8109–8115.

Mitchison, T.J. (2012). The proliferation rate paradox in antimitotic chemotherapy. Mol. Biol. Cell *23*, 1–6.

Muellner, M.K., Uras, I.Z., Gapp, B.V., Kerzendorfer, C., Smida, M., Lechtermann, H., Craig-Mueller, N., Colinge, J., Duernberger, G., and Nijman, S.M. (2011). A chemical-genetic screen reveals a mechanism of resistance to PI3K inhibitors in cancer. Nat. Chem. Biol. *7*, 787–793.

Murai, J., Huang, S.Y., Das, B.B., Renaud, A., Zhang, Y., Doroshow, J.H., Ji, J., Takeda, S., and Pommier, Y. (2012). Trapping of PARP1 and PARP2 by clinical PARP inhibitors. Cancer Res. *72*, 5588–5599.

Murai, J., Huang, S.Y., Renaud, A., Zhang, Y., Ji, J., Takeda, S., Morris, J., Teicher, B., Doroshow, J.H., and Pommier, Y. (2014). Stereospecific PARP trapping by BMN 673 and comparison with olaparib and rucaparib. Mol. Cancer Ther. *13*, 433–443.

Norquist, B., Wurz, K.A., Pennil, C.C., Garcia, R., Gross, J., Sakai, W., Karlan, B.Y., Taniguchi, T., and Swisher, E.M. (2011). Secondary somatic mutations restoring BRCA1/2 predict chemotherapy resistance in hereditary ovarian carcinomas. J. Clin. Oncol. *29*, 3008–3015.

Olaussen, K.A., Dunant, A., Fouret, P., Brambilla, E., André, F., Haddad, V., Taranchon, E., Filipits, M., Pirker, R., Popper, H.H., et al.; IALT Bio Investigators (2006). DNA repair by ERCC1 in non-small-cell lung cancer and cisplatin-based adjuvant chemotherapy. N. Engl. J. Med. *355*, 983–991.

Pommier, Y., Leo, E., Zhang, H., and Marchand, C. (2010). DNA topoisomerases and their poisoning by anticancer and antibacterial drugs. Chem. Biol. *17*, 421–433.

Shen, Y., Rehman, F.L., Feng, Y., Boshuizen, J., Bajrami, I., Elliott, R., Wang, B., Lord, C.J., Post, L.E., and Ashworth, A. (2013). BMN 673, a novel and highly potent PARP1/2 inhibitor for the treatment of human cancers with DNA repair deficiency. Clin. Cancer Res. *19*, 5003–5015.

Shen, J., Peng, Y., Wei, L., Zhang, W., Yang, L., Lan, L., Kapoor, P., Ju, Z., Mo, Q., Shih, I.-M., et al. (2015). ARID1A deficiency impairs the DNA damage checkpoint and sensitizes cells to PARP inhibitors. Cancer Discov. *5*, 752–767.

Subramanian, A., Tamayo, P., Mootha, V.K., Mukherjee, S., Ebert, B.L., Gillette, M.A., Paulovich, A., Pomeroy, S.L., Golub, T.R., Lander, E.S., and Mesirov, J.P. (2005). Gene set enrichment analysis: a knowledge-based approach for interpreting genome-wide expression profiles. Proc. Natl. Acad. Sci. USA *102*, 15545–15550.

Swisher, E.M., Lin, K.K., Oza, A.M., Scott, C.L., Giordano, H., Sun, J., Konecny, G.E., Coleman, R.L., Tinker, A.V., O'Malley, D.M., et al. (2017). Rucaparib in relapsed, platinum-sensitive high-grade ovarian carcinoma (ARIEL2 Part 1): an international, multicentre, open-label, phase 2 trial. Lancet Oncol. *18*, 75–87.

Yokoyama, Y., Matsushita, Y., Shigeto, T., Futagami, M., and Mizunuma, H. (2014). Decreased ARID1A expression is correlated with chemoresistance in epithelial ovarian cancer. J. Gynecol. Oncol. *25*, 58–63.

Cell Reports, Volume *23*

Supplemental Information

A Quantitative Chemotherapy Genetic Interaction Map Reveals Factors Associated with PARP Inhibitor Resistance

Hsien-Ming Hu, Xin Zhao, Swati Kaushik, Lilliane Robillard, Antoine Barthelet, Kevin K. Lin, Khyati N. Shah, Andy D. Simmons, Mitch Raponi, Thomas C. Harding, and Sourav Bandyopadhyay

SUPPLEMENTAL FIGURES

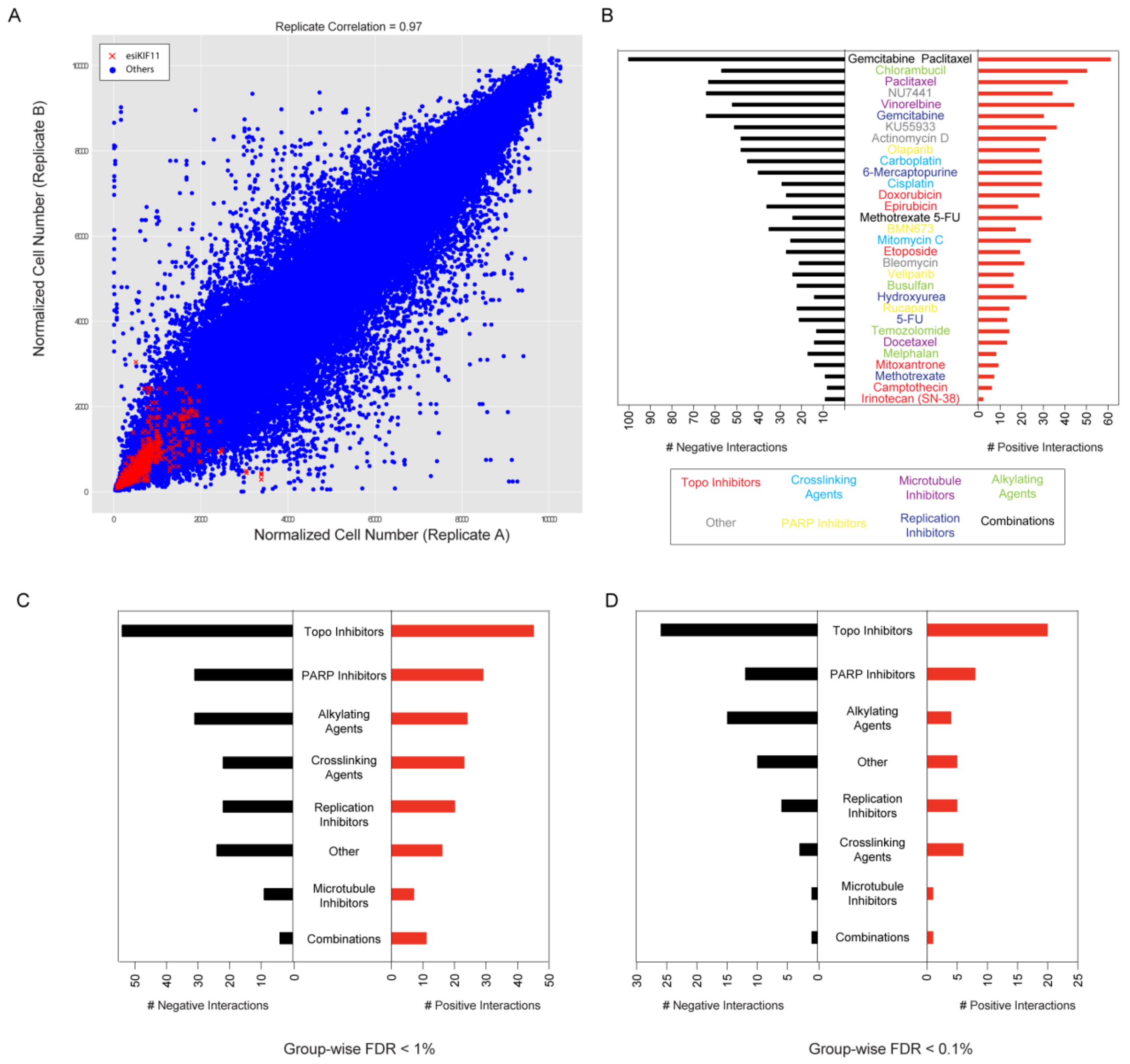

Figure S1. Properties of the chemical-genetic interaction map, Related to Figure 1 and 2. (A) Comparison of cell number between technical replicates shown. Red crosses indicate cell numbers in the context of knockdown of the essential gene KIF11. Pearson correlation among replicates shown. **(B)** Number of high confidence interaction per drug. Interactions are considered if they have a score greater than 3 (positive) or less than -3 (negative) corresponding to a 1% FDR. **(C-D)** Number of interactions based on common interactions with drugs in the same class. The median of interactions of a given gene with all drugs in a class was compared to a permuted background to derive a False Discovery Rate (FDR). Number of interactions at an FDR (C) less than 1% or (D) less than 0.1%

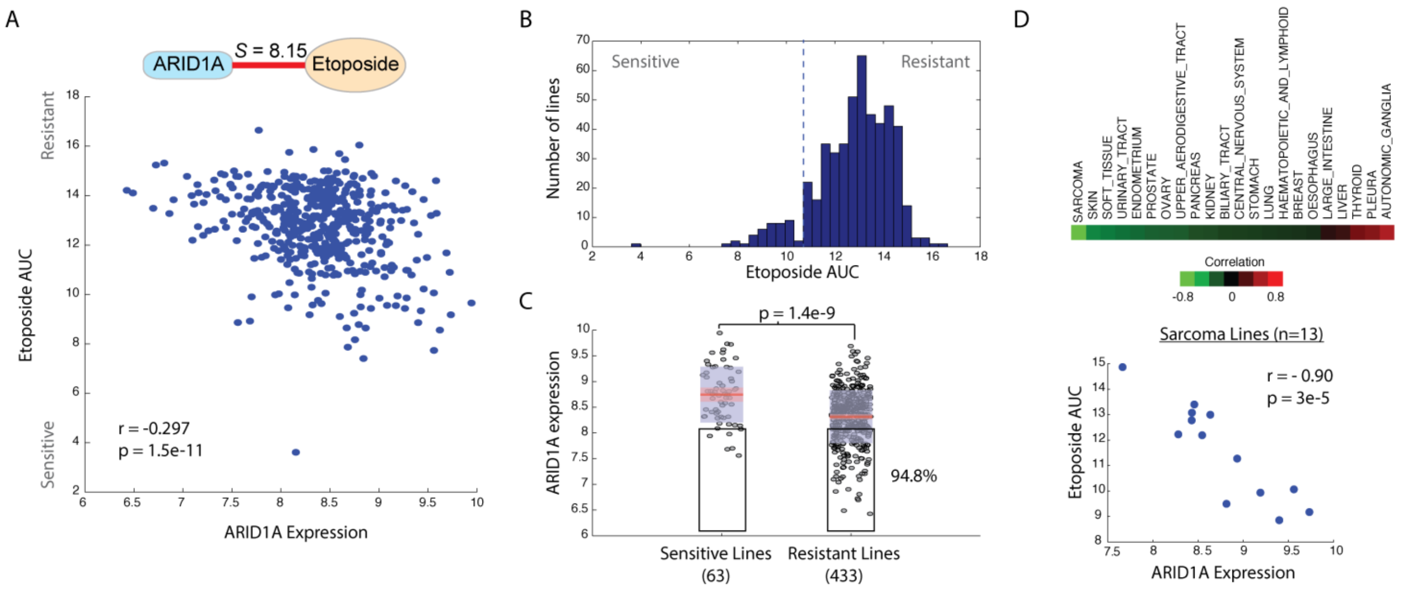

Figure S2. ARID1A loss is associated with etoposide resistance as predicted by the map, Related to Figure 2. (A) Correlation of ARID1A expression and etoposide sensitivity across 496 cancer cell lines based on area under the curve (AUC) analyis of drug sensitivity in the CTRP dataset. **(B)** Histogram of sensitivity to Etoposide across 496 lines. Dotted line represents an AUC of 11 which is used to separate 63 sensitive versus 433 resistant lines based on an approximately bimodal distribution. **(C)** ARID1A expression in sensitive versus resistant lines. Lines with expression < 8 are outlined and 94.8% of the cell lines that have this level of expression or lower are considered drug resistant. **(D)** Correlation analysis of ARID1A expression with etoposide AUC across subsets of cell lines with the indicated lineages. Scatter plot of expression and AUC for sarcoma lines shown. P-values based on pearson correlation except for (C) where it is based on a two-tailed t-test. Box plots are medians +/- s.d.

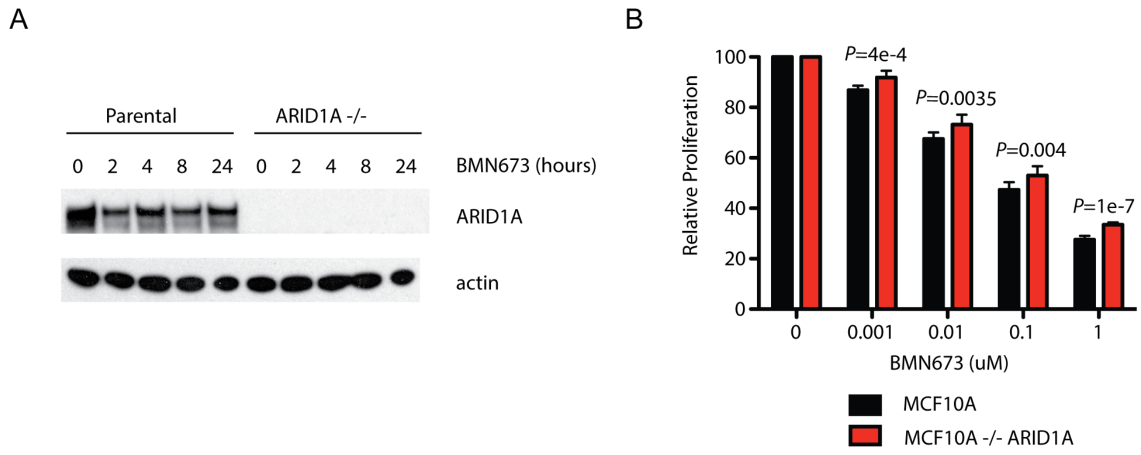

Figure S3. ARID1A deletion leads to PARP inhibitor resistance, Related to Figure 5. (A) Verification of ARID1A loss in isogenic MCF10A parental and ARID1A -/- cells by immunoblot. **(B)** Sensitivity to BMN673 of ARID1A null cells compared to parental MCF10A.

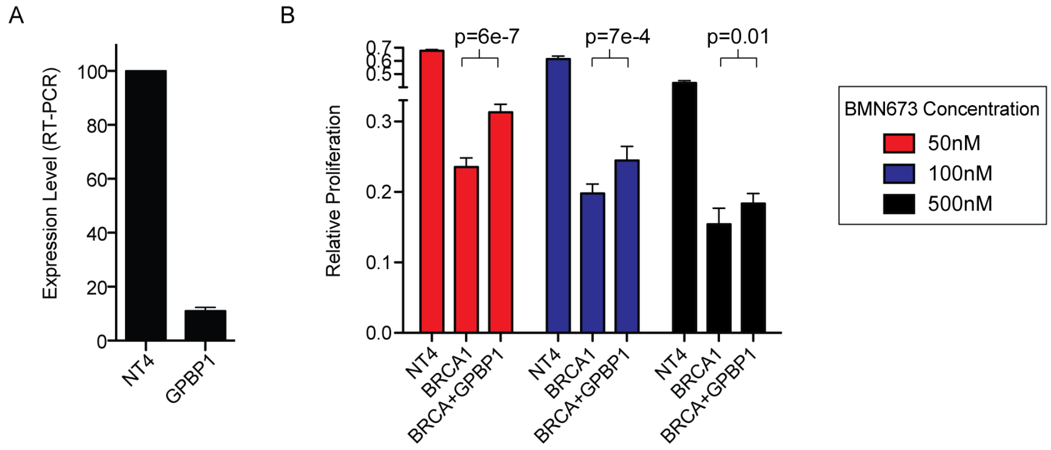

Figure S4. GPBP1 knockdown and effects on BMN673 sensitivity, Related to Figure 6. (A) RNA from MCF10A cells transfected with either siNT4 or siGPBP1 was subjected to RT-PCR analysis for GPBP1 expression. **(B)** Proliferation of MCF10A cells trasnfected with siRNAs targeting the indicated gene or non-targeting (NT4) and then treated with the indicated dose of BMN673 for 3 days.

SUPPLEMENTAL EXPERIMENTAL PROCEDURES

Immunofluorescence microscopy

MCF10A cells were grown on glass coverslip pre-coated with polylysine. To induce DNA damage repair foci the cells were treated with 0.5mM BMN673 for 4 or 8 hours. Cells were fixed with 4% paraformaldehyde in PBS for 10 min, permeablized with 0.3% Triton for 10 min, and blocked with 3% BSA in PBS for 40 min at room temperature. Cells were then incubated with antibodies against gH2AX (JBW301, Upstate) or RAD51 (SC-8349, Santa Cruz) at 4°C overnight, followed by the incubation with species-specific Alexa488 or Alexa647-conjugated secondary antibodies (Thermofisher) for 1hr at room temperature. Coverslips were mounted on the slides in Vectashield containing DAPI for nuclear counterstain. Images were acquired with a confocal microscope (Zeiss LSM 780) equipped with a 63x water immersion objective and a CCD camera, using the ZEN software. Automated foci counting was done using the Foci Counter (The Bioimaging Center, University of Konstanz) plugin in ImageJ. At least 150 cells were scored for each experiment, and each experiment was repeated three times.

siRNA transfection

SMARTpool ON-TARGETplus siRNAs targeting BRCA1(L-003461-00-0005), ARID1A (L-017263-00-0005), GPBP1 (L-014236-02-0005) and non-targeting control siNT4 were purchased from GE Dharmacon. siRNAs were transfected into cells using Lipofectamine RNAiMax transfection reagent (ThermoFisher) according to the manufacturer's instruction. The cells were transfected in 384-well plates at a final siRNA concentration of 20nM. After an initial 24h period of gene knockdown, drug sensitivity was determined by adding the indicated drugs for 72h.

Drug combination screening

Cells were seeded onto 384-well plates (1,000 cells/well) 18 hours prior to treatment with drug either singly or in combination with another drug. A pair of drugs is combined in a series of one DMSO and five

or seven concentrations centered on the IC_{50} dose, resulting in a 6 × 8 escalating combination dose matrix using a robotic liquid handler. After a 72 hour drug treatment, cell proliferation was determined by staining with Hoescht 33342 (5ug/ml) and cell number counted using a Thermo CellInsight High Content microscope. Synergistic effects between different drug combos was measured by determination of synergy score using comparison to Loewe additive model with Chalice Analyzer (Zalicus Inc. Cambridge, MA). Combination Index values were calculated using CompuSyn (www.compusyn.com).

Cell line drug sensitivity prediction

A total of 568 genes were mapped between the chemical-genetic interaction map and gene expression data obtained from (https://portals.broadinstitute.org/ccle/home). Expression data was normalized to a median of 0 across cell lines. Sensitivity for each drug was defined by its area under the curve, or AUC (Basu et al., 2013). To predict the sensitivity of a given cell line for a particular drug in an unsupervised fashion, the sign of the chemical-genetic interaction score for each gene in the map was multiplied by the normalized expression of that gene and this product summed over all genes in the map. Hence, for each cell line the predicted AUC for drug *d* ($pAUC_d$) is:

$$pAUC_d = -\sum_{g \in G} e_g * sign(s_{gd})$$

Where G is the set of all genes in the map for drug d, s_{gd} is the score of drug d with gene g and e_g is the normalized expression of g in this cell line. This procedure rewards for cases where the expression and interaction scores are consistent, i.e. when both expression and score is negative or both expression and score are positive, and in both these cases will predict drug sensitivity. This procedure was performed at differing cutoffs of the interaction map based on the absolute value of the score. For each drug a *pAUC* was computed for each cell line and this value was then correlated with the published AUC.

Reverse transcription PCR (RT-qPCR)

2.5 mg of total RNA was reverse transcribed using murine Moloney leukemia virus (MMLV) reverse transcriptase, with oligo dT and template switching oligos as primers for cDNA synthesis. Real-time PCR with SYBR green detection was performed using an ABI Prism 7700 thermocycler (Applied Biosystem). The house keeping genes HPRT-1 and b2M was used as control for normalization. A ΔΔCq method was used to process the data for the target gene (GPBP1) to calculate relative gene expression normalized to house keeping genes.

RNAseq analysis

RNA extraction from MCF10A cells was performed with RNeasy mini kit (Qiagen). The samples were treated sequentially with Baseline-ZERO DNase and Ribo-ZERO RNA removal kit (Epicentre) to deplete DNA and rRNA. 35ng of rRNA-depleted sample was used as input for library preparation using ScriptSeq v2 library preparation kit (Epicentre) according to the manufacturer's instructions. All libraries were indexed with Illumina barcodes for multiplexing. The quality of the library preparations was assessed on the 2100 Bioanalyzer (Agilent) using a High Sensitivity DNA Chip. The quantitation of the libraries was performed using the Qubit dsDNA HS Assay kit (Life technology). Sequencing was performed on an Illumina HiSeq 4000 for single-ended 50 base paired reads in duplicate on Illumina Hi-Seq 2000 at the Center for Advanced Technology at the University of California, San Francisco. Raw sequencing reads were analyzed using FASTQC for quality control and adapters were filtered using cutadapt (Martin, 2017). The filtered raw reads were pseudoaligned and quantification of the transcripts was performed with Kallisto (Bray et al., 2016) using GRC38 reference human transcriptome. Transcript abundance estimates were converted to gene level estimates using an R package Tximport (Soneson et al., 2015). Differential expression analysis between sets of conditions was performed using DESeq2 and averaged over both replicates (Anders and Huber, 2010). Analysis of the enrichment of functional gene groups and pathways among differentially expressed genes was performed using gene-set enrichment analysis (GSEA) (Subramanian et al., 2005). Enrichment plots were calculated based on ranking of differentially

expressed genes between different conditions. RNA-seq data are available under GEO:GSE101904 (https://www.ncbi.nlm.nih.gov/geo/).

Clinical trial data analysis

Tumor samples were tested for mutations in a panel of 322 genes using FoundationOne test from Foundation Medicine (Waltham, MA). In the ARIEL-2 trial (NCT01891344) 154 patients were selected that were wild type in HR pathways genes including *BRCA1*, *BRCA2* and *CDK12*. Clinical characteristics were taken from Swisher et al (Swisher et al., 2017). ARIEL-2 was approved by the institutional review board at each study site and was done in accordance with the Declaration of Helsinki and Good Clinical Practice Guidelines of the International Conference on Harmonisation. Patients provided written informed consent before participation.

Statistical analysis

All error bars are s.d. unless otherwise noted. All P-values based on two-tailed t-test.

Supplemental References

Anders, S., and Huber, W. (2010). Differential expression analysis for sequence count data. Genome biology *11*, R106.

Basu, A., Bodycombe, N.E., Cheah, J.H., Price, E.V., Liu, K., Schaefer, G.I., Ebright, R.Y., Stewart, M.L., Ito, D., Wang, S., *et al.* (2013). An interactive resource to identify cancer genetic and lineage dependencies targeted by small molecules. Cell *154*, 1151-1161.

Bray, N.L., Pimentel, H., Melsted, P., and Pachter, L. (2016). Near-optimal probabilistic RNA-seq quantification. Nature biotechnology *34*, 525-527.

Martin, M. (2017). Cutadapt removes adapter sequences from high-throughput sequencing reads. EMBnet.

Soneson, C., Love, M.I., and Robinson, M.D. (2015). Differential analyses for RNA-seq: transcript-level estimates improve gene-level inferences. F1000Research *4*, 1521.

Subramanian, A., Tamayo, P., Mootha, V.K., Mukherjee, S., Ebert, B.L., Gillette, M.A., Paulovich, A., Pomeroy, S.L., Golub, T.R., Lander, E.S., *et al.* (2005). Gene set enrichment analysis: a knowledge-based approach for interpreting genome-wide expression profiles. Proceedings of the National Academy of Sciences of the United States of America *102*, 15545-15550.

Swisher, E.M., Lin, K.K., Oza, A.M., Scott, C.L., Giordano, H., Sun, J., Konecny, G.E., Coleman, R.L., Tinker, A.V., O'Malley, D.M., *et al.* (2017). Rucaparib in relapsed, platinum-sensitive high-grade ovarian carcinoma (ARIEL2 Part 1): an international, multicentre, open-label, phase 2 trial. The Lancet Oncology *18*, 75-87.
